# Self-Assembly of Tert-Butyl-Substituted Pentaphosphaferrocenes with Copper Halides: Selective Formation of One- and Two-Dimensional Coordination Polymers

**DOI:** 10.3390/molecules31142507

**Published:** 2026-07-17

**Authors:** Bijan Mondal, Mehdi Elsayed Moussa, Michael Seidl, Manfred Scheer

**Affiliations:** Department of Inorganic Chemistry, University of Regensburg, 93040 Regensburg, Germany; mondal.bijan@gmail.com (B.M.); mehdi.elsayed-mousa@chemie.uni-regensburg.de (M.E.M.); michael.seidl@uibk.ac.at (M.S.)

**Keywords:** copper halides, coordination polymers, cyclopentadienyl ligands, mixed linkers, self-assembly

## Abstract

Over the past three decades, pentaphosphaferrocene [Cp*Fe(η^5^-P_5_)] (1*) has emerged as a versatile building block in coordination chemistry, enabling the construction of a wide range of nanosized spherical aggregates and extended coordination polymers (CPs). Previous studies have shown that modification of substituents in fully substituted cyclopentadienyl ligands strongly influences the size of the resulting spherical aggregates. However, the impact of partially substituted cyclopentadienyl ligands remains unexplored. Herein, we investigate the reactivity of the *cyclo*-P_5_ ligands in tert-butyl-substituted pentaphosphaferrocenes [Cp^R^Fe(η^5^-P_5_)] {Cp^R^ = Cp″: η^5^-1,3-(^t^Bu)_2_C_5_H_3_ (**1″**) and Cp**‴**: η^5^-1,2,4-(^t^Bu)_2_C_5_H_2_ (**1‴**)} towards Cu(I) halides (CuX; X = Cl, Br and I). These reactions yield a series of CPs (**2**–**9**) that are structurally characterized by single-crystal X-ray diffraction, multinuclear NMR spectroscopy, and ESI-MS spectrometry. Reactions of **1″** with CuCl and CuBr afford one-dimensional CPs while reactions with CuI show a pronounced dependence on stoichiometry, leading to CPs with different structural topologies and dimensionalities. In contrast, the bulkier derivative **1‴** consistently forms two-dimensional networks. These include a regular sheet-like framework (**7**), an architecture composed of alternating mesh sizes (**8**), and a distinctive double-layered structure (**9**) featuring Cu_4_I_3_ and Cu_4_I_4_ nodes interconnected by organometallic (**1‴**) and inorganic (CuI_2_) linkers. Solution studies indicate partial depolymerization of the polymers, consistent with the lability of Cu-P interactions.

## 1. Introduction

Over the past few decades, coordination polymers (CPs) have evolved from a niche research topic into one of the most dynamic and rapidly expanding areas in modern chemistry [1,2,3,4]. This development is driven not only by their virtually unlimited structural diversity and fascinating topological architectures, but also by their broad range of potential applications, particularly in energy-related processes and gas storage [5,6,7,8,9,10]. The significance of CP chemistry is perhaps most prominently highlighted by metal–organic frameworks (MOFs), a class of porous 3D CPs, whose development was recognized by the 2025 Nobel Prize in Chemistry [11]. The vast majority of CPs are constructed via metal-directed self-assembly, employing tailor-made multitopic organic molecules (most commonly bearing N- or O-donor atoms) that coordinate to metal ions [12,13]. In addition to simple metal centers, metal clusters have occasionally been used as nodes, providing access to novel materials with pronounced semiconducting and magnetic properties [14,15]. By comparison, organometallic building blocks have only rarely been utilized as linkers in CP chemistry [16,17,18,19]. In this context, our group introduced the concept of using polyphosphorus (P*_n_*) ligand complexes as versatile organometallic linkers for connecting metal centers [20]. Owing to their highly flexible coordination behavior, these complexes enable the formation of a broad spectrum of supramolecular architectures, ranging from discrete aggregates (including nanosized spherical assemblies) to extended 1D, 2D and 3D CPs.

**Figure 1:**
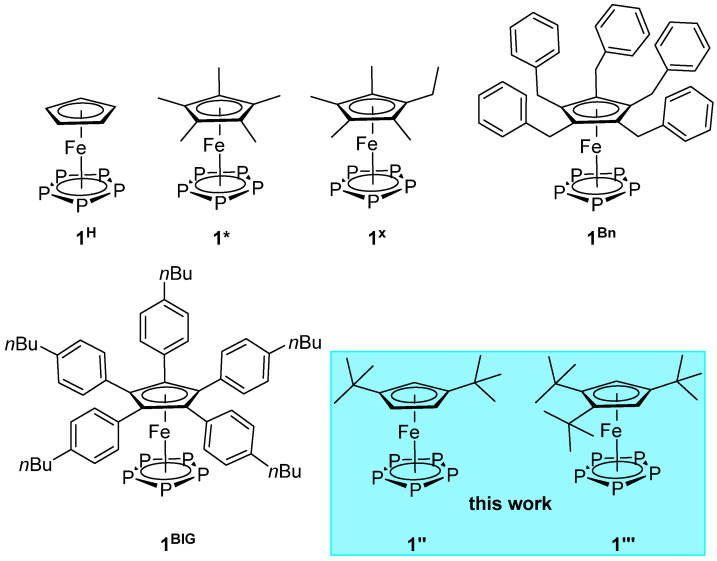
Pentaphosphaferrocene derivatives **1^H^**, 1*, **1^X^**, **1^BIG^** and **1^Bn^** previously used in metal-directed self-assembly and derivatives **1**″ and **1‴** presented in this work.

Among these systems, the pentaphosphaferrocene derivative [Cp*Fe(η^5^-P_5_)] (1*, Figure 1) [21] has emerged as the most extensively investigated building block, particularly in combination with group 13 and coinage metal salts [20,22]. A notable example is its reactions with copper halides. Under concentrated reaction conditions (typically > 15 mmolL^−1^), these reactions afford a variety of insoluble 1D and 2D CPs [23]. In striking contrast, under diluted reaction conditions (<15 mmolL^−1^), similar reactions lead exclusively to the formation of soluble nanosized spherical aggregates [20] clearly demonstrating the sensitivity of the self-assembly process to external parameters. More recently, the influence of substituents on the cyclopentadienyl ligand (Cp^R^) in pentaphosphaferrocene derivatives of the type [Cp^R^Fe(η^5^-P_5_)] (Cp^R^ = C_5_H_5_ (Cp), (**1^H^**); C_5_Me_4_Et (Cp^x^), (**1^X^**); C_5_(CH_2_C_6_H_5_)_5_ (Cp^Bn^), and C_5_(C_6_H_4_-4-*^n^*Bu)_5_ (Cp^BIG^)) has been systematically examined. For example, the reaction of **1^H^** with copper halides exclusively yielded CPs, with no spherical supramolecules observed under any reaction conditions [24], a behavior attributed to the low solubility of **1^H^** compared to the other derivatives in Figure 1. In contrast, derivative **1^X^** exhibits coordination behavior closely resembling that of 1* towards copper salts.

Both compounds react with CuBr to selectively form fullerene-like nanoballs of the general composition [{Cp^R^Fe(η^5^:η^1^:η^1^:η^1^:η^1^:η^1^-P_5_)}_12_{CuBr}_10_{Cu_2_Br_3_}_5_{Cu(CH_3_CN)_2_}_5_] (Cp^R^ = C_5_Me_5_, C_5_Me_4_Et), featuring cluster cores of 90 non-carbon atoms [25]. Even more pronounced substituent effects are observed for the bulky derivatives **1^Bn^** and **1^BIG^**, which not only display a strong tendency to form spherical aggregates upon coordination to copper salts, but also tend to generate significantly larger supramolecular assemblies than those derived from 1* [26,27,28]. For instance, analogous reactions of 1* and **1^Bn^** with Cu(CF_3_SO_3_)·0.5C_7_H_8_ under comparable reaction conditions yield fundamentally different products: while 1* selectively forms the 2D polymer [{Cp*Fe(ƞ^5:1:1:1^-P_5_)}{Cu(CF_3_SO_3_)}]_n_, derivative **1^Bn^** affords the spherical aggregate [{Cp^Bn^Fe(ƞ^5^-P_5_)}_12_{Cu(CF_3_SO_3_)}_19_._6_] [29]. These findings clearly demonstrate that the nature of the Cp^R^ ligand is a decisive factor governing whether coordination to copper salts result in discrete spherical assemblies or extended CPs. Motivated by these observations, we sought to explore pentaphosphaferrocene derivatives featuring Cp^R^ ligands that differ fundamentally from those investigated previously. Herein, we report the coordination chemistry of the tert-Bu substituted derivatives [Cp″Fe(η^5^-P_5_)] (**1**″) and [Cp‴Fe(η^5^-P_5_)] (**1**‴) with copper halides under various reaction conditions and compare their behavior with that of 1*. In contrast to previously studied systems, these compounds lack five-fold symmetry at the Cp^R^ ligand. Moreover, whereas the substituents in **1^Bn^** [30] and **1^BIG^** [31] are oriented fully or partially, respectively, out of the Cp plane, the tert-Bu groups in both **1**″ [32] and **1**‴ [32] are located predominantly within the Cp plane. As a consequence, these derivatives exclusively yield 1D and 2D CPs with various distinct structural features, with no evidence for the formation of spherical aggregates, irrespective of concentration or stoichiometry. The detailed results of these investigations are presented herein.

## 2. Results and Discussion

### 2.1. Self-Assembly of [Cp″Fe(η^5^-P_5_)] (**1″**) with CuX (X = Cl, Br, I)

**Scheme 1:**
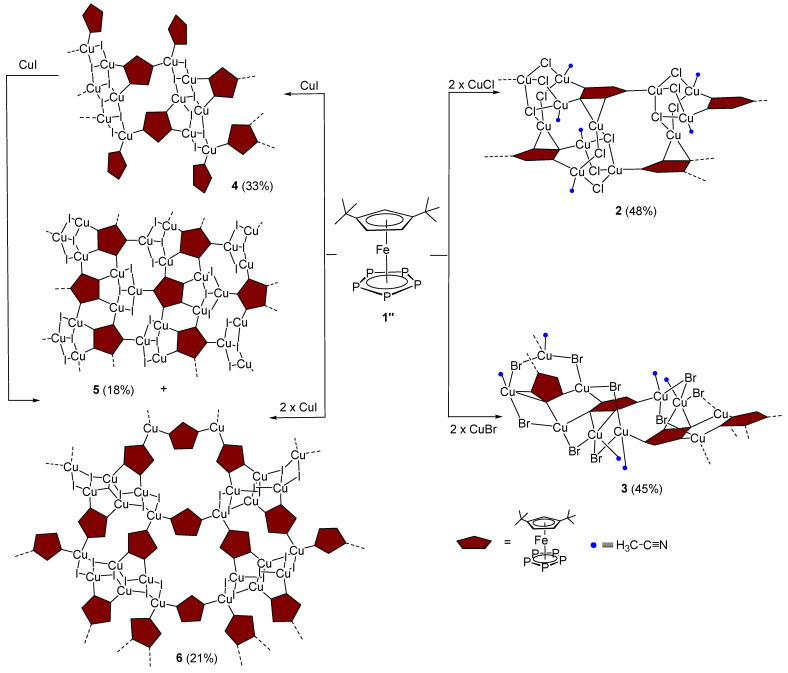
Self-assembly of **1**″ with CuX (X = Cl, Br and I). Schematic presentation of sections of the formed CPs **2**–**6**; isolated yields are given in parenthesis.

**Figure 2:**
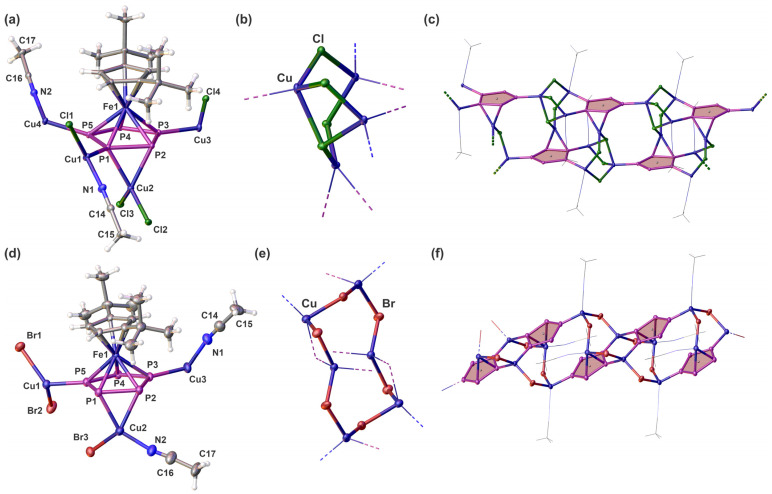
Asymmetric units of compounds (**a**) **2** and (**d**) **3**; disorders and solvent molecules have been omitted for clarity. Copper halide nodes of (**b**) **2** and (**e**) **3**; sections of their 1D polymeric frameworks (**c**) **2** and (**f**) **3**; selected bond lengths (Å) for **2**: Cu3–Cl4 2.2805(5), Cu3–P3 2.1922(6), Cu1–P1 2.1993(6), Cu1–N1 1.9820(18), Cu1–Cl1 2.3020(5), P2–P1 2.1697(7), P2–P3 2.1336(7), Cu2–P1 2.3666(6) Cu2–P2 2.3400(6), Fe1–P3 2.3459(6), Fe1–P1 2.3760(6). Selected bond lengths (Å) for **3**: Cu1–P1 2.2395(15), Cu2–P2 2.3537(14), Cu2–P3 2.4304(16), Br1–Cu1 2.4473(9), Cu2–N2 1.994(5), Fe1–P1 2.3279(15), P1–P2 2.1149(17), P1–P5 2.0961(19). Extended polymeric network/connection is shown by the dotted lines.

In an initial approach, [Cp″Fe(η^5^-P_5_)] (**1**″) was reacted with copper halides following a procedure analogous to that reported for 1* and copper halides [23]. In a typical experiment, a CH_2_Cl_2_ solution of **1**″ (1 equiv.) was carefully layered with a 1:1 CH_2_Cl_2_/CH_3_CN mixture, which was subsequently overlaid with a CH_3_CN solution of CuCl or CuBr (2 equiv.). After complete diffusion and homogenization of the phases, no crystalline material was initially observed. However, in both cases the reaction mixture turned brown, in contrast to the green color of the starting material pentaphosphaferrocene **1**″. Subsequent layering of the crude mixture with *n*-hexane (3-fold excess) afforded red crystals of **2** (from **1**″ and CuCl) and brown crystals of **3** (from **1**″ and CuBr) in good yields (Scheme 1). Compounds **2** and **3** crystallize in the monoclinic space group *P*2_1_/*n* and the triclinic space group *P*$\bar{1}$, respectively. Single crystal X-ray structure analyses reveal 1D CPs of the general formulas [{Cp″Fe(μ,η^5^:η^2^:η^1^:η^1^:η^1^-P_5_)}(CuCl)_4_(CH_3_CN)_2_]_∞_ (**2**) and [{Cp″Fe(μ,η^5^:η^2^:η^1^:η^1^-P_5_)}(CuBr)_3_(CH_3_CN)_2_]_∞_ (**3**), exhibiting 1,1,2,3,5- and 1,2,3,5-coordination modes, respectively, of the *cyclo*-P_5_ ligand (Figure 2, Table 1). Both polymers display unprecedented structural motifs within this class of compounds.

Polymer **2** consists of two parallel 1D strands composed of alternating *cyclo*-P_5_ linkers and Cu_4_Cl_4_(CH_3_CN)_2_ nodes, resulting in a double-stranded CP (Table 1, Figure 2c). Each Cu_4_Cl_4_(CH_3_CN)_2_ unit contains four tetrahedrally distorted Cu(I) centers, linked by three μ_2_-Cl and one μ_3_-Cl ligand (Figure 2b). Within each node, three Cu centers propagate the same strand via single Cu-P interactions with two different P_5_ ligands. The fourth Cu center establishes interstrand connectivity by coordinating in an η^2^-fashion to two adjacent P atoms of a *cyclo*-P_5_ ligand belonging to the neighboring strand.

Consequently, each Cu_4_Cl_4_(CH_3_CN)_2_ unit simultaneously mediates intra-strand propagation and inter-strand crosslinking, giving rise to a double-stranded architecture. In contrast, polymer **3** adopts a single-stranded 1D structure based on repeating Cu_6_Br_6_(P_5_)_2_ units (Scheme 1, Figure 2f). The core structural motif is a Cu_6_Br_6_ metallacycle composed of six Cu(I) centers bridged by six μ_2_-Br ligands (Figure 2e). The four peripheral Cu(I) atoms are coordinated by terminal acetonitrile ligands, whereas the two central Cu(I) sites remain uncoordinated by solvent molecules. Each Cu_6_Br_6_ unit is associated with two *cyclo*-P_5_ ligands positioned on opposite sides of the metallacyclic plane. Within one repeating fragment, each P_5_ ligand engages in a mixed coordination mode toward three Cu centers of the same Cu_6_Br_6_ core: two adjacent P atoms (P1, P2) bind in an η^2^-fashion to a terminal CuBr(CH_3_CN) fragment, while P1 and P5 each coordinate in an η^1^-mode to central Cu atoms. In addition, P3 forms an η^1^ Cu–P bond to a terminal CuBr(CH_3_CN) unit of a neighboring Cu_6_Br_6_ metallacycle, through which polymer propagation occurs (Figure 2d,e).

Each *cyclo*-P_5_ ligand thus contributes one interunit η^1^ Cu–P linkage and four such connections between adjacent Cu_6_Br_6_(P_5_)_2_ fragments generate the extended 1D chain (Table 1, Figure 2e). Notably, polymers **2** and **3** are selectively obtained regardless of whether 1:1, 1:2 or 2:1 stoichiometric ratios of the starting materials are employed, although the 1:2 ratio of [Cp″Fe(η^5^-P_5_)] to CuX affords the highest crystalline yields.

**Figure 3:**
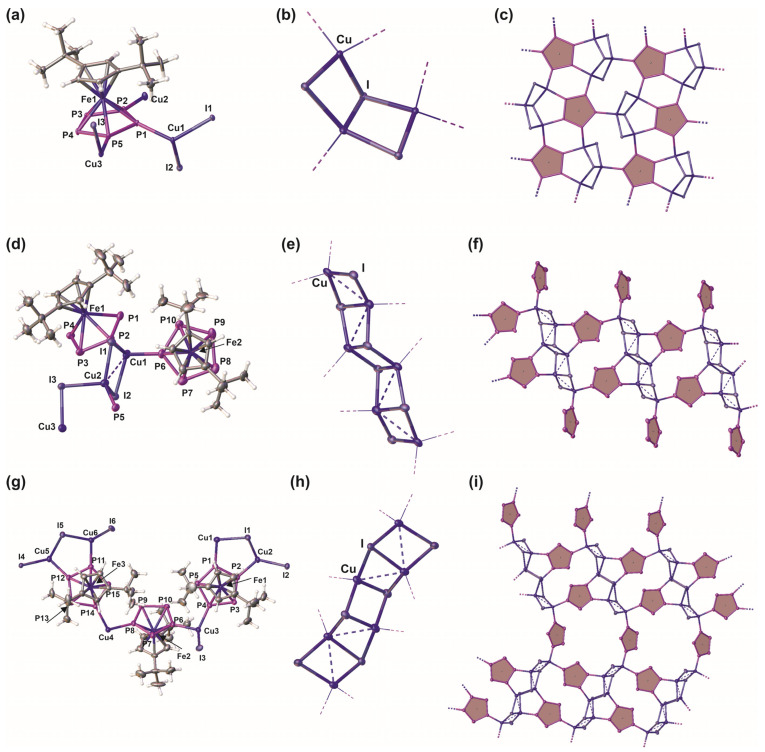
Asymmetric units of compounds (**a**) **4**, (**d**) **5** and (**g**) **6**; disorders and solvent molecules have been omitted for clarity. Copper halide nodes of (**b**) **4**, (**e**) **5** and (**h**) **6**; sections of their polymeric frameworks (**c**) **4**, (**f**) **5** and (**i**) **6**; selected bond lengths (Å) for **4**: Cu1–Cu2 2.9893(18), Cu2–Cu3 3.0152(14), Cu1–l1 2.5982(12), Cu1–l2 2.7549(12), Cu2–l1 2.6069(11), Cu2–l2 2.6564(12), Cu1–P2 2.2772(19), Cu1–P6 2.203(7), Cu2–P5 2.270(2), P2–P1 2.114(3), P2–P3 2.107(3), P6–P7 2.116(7), P6–P10 2.096(8), Fe1–P3 2.4238(19), Fe1–P1 2.401(2), Fe2–P6 2.358(7), Fe2–P9 2.371(6). Selected bond lengths (Å) for **5**: Cu1–l1 2.6148(10), Cu1–l2 2.5724(11), Cu1–P1 2.283(2), Cu2–P2 2.2555(18), Cu3–P5 2.299(2), Cu3–I3 2.5702(10), Fe1–P5 2.372(2), Fe1–P1 2.395(2), Fe1–P2 2.409(2). Selected bond lengths (Å) for **6**: Cu1–l1 2.7206(15), Cu2–l1 2.6512(15), Cu2–l2 2.5997(14), Cu1–P1 2.274(2), Cu2–P2 2.274(2), Cu3–P4 2.270(2), Cu3–P6 2.274(2), Cu3–I3 2.499(4), Cu4–P14 2.270(2), Cu4–P8 2.278(2), Cu5–l4 2.565(6), Cu5–l5 2.6454(14), Cu5–P12 2.276(2), Cu6–l5 2.6939(14), Cu6–l6 2.5886(14), Cu6–P11 2.274(2), P1–P2 2.104(3), P6–P10 2.106(4), P6–P7 2.101(3), P13–P14 2.105(3), P11–P12 2.103(3), Fe1–P1 2.360(3), Fe1–P5 2.393(3), Fe2–P8 2.338 (2), Fe3–P14 2.354(3). Extended polymeric network/connection is shown by the dotted lines.

In contrast, the reaction of [Cp″Fe(η^5^-P_5_)] (**1**″) with CuI was found to be dependent on the stoichiometric ratio of the reactants. For example, employing a 1:1 ratio enabled the selective isolation of polymer **4** of the general formula [{Cp″Fe(μ,η^5^:η^1^-P_5_)}({Cp″Fe(μ,η^5^:η^1^:η^1^:η^1^-P_5_)}(Cul)_3_]_∞_ (Scheme 1). However, the 1:2 ratio of **1**″:CuI resulted in the formation of polymers [{Cp″Fe(μ,η^5^:η^1^:η^1^:η^1^:η^1^:η^1^-P_5_)}(Cul)_3_]_∞_ (**5**) and [{Cp″Fe(μ,η^5^:η^1^:η^1^-P_5_)}({Cp″Fe(μ,η^5^:η^1^:η^1^:η^1^-P_5_)}_2_(Cul)_6_]_∞_ (**6**), respectively (Scheme 1), which can be manually separated from one another due to their difference in color. Compounds **4**–**6** crystallize in the triclinic space group *P*$\bar{1}$ (**4**,**6**) and the orthorhombic space group *P*2_1_2_1_2_1_ (**5**). Interestingly, whereas the reactions with CuCl and CuBr exclusively yield 1D CPs, single crystal X-ray diffraction analysis reveals 1D topology for **4** and 2D architectures for **5** and **6**. Polymer **4** is constructed from Cu_6_I_6_ cluster nodes interconnected by *cyclo*-P_5_ ligands (Figure 3c). The asymmetric unit comprises three Cu(I) centers, three iodide ions and two *cyclo*-P_5_ ligands, while the complete Cu_6_I_6_ cluster node is generated by crystallographic symmetry (Figure 3a). Structurally, the Cu_6_I_6_ unit can be described as consisting of two parallel strands composed of alternating Cu and I atoms (Cu–I–Cu–I–Cu–I). These strands are arranged in an antiparallel fashion such that each Cu atom of one strand is positioned opposite an I atom of the neighboring strand. This arrangement results in a Cu_6_I_6_ core composed of five fused Cu_2_I_2_ four-membered rings (Figure 3b). Within these clusters, short Cu···Cu separations in the range 2.989 to 3.072 Å indicate the possible presence of weak cuprophilic interactions [33]. Each Cu_6_I_6_ node is coordinated by two *cyclo*-P_5_ ligands located on opposite sides of the cluster core. These ligands bind to the node through a single P atom in an η^1^ coordination mode. In addition, each pair of adjacent nodes is bridged by two *cyclo*-P_5_ ligands acting as multidentate linkers that coordinate through three P atoms to copper centers of neighboring cluster units. As a result, the Cu_6_I_6_ nodes are interconnected through pairs of *cyclo*-P_5_ bridges, giving rise to a ladder-like 1D CP (Figure 3c). The asymmetric unit of **5** contains one *cyclo*-P_5_ ligand coordinated through three P atoms to Cu–I fragments (Figure 3d). The metal nodes consist of Cu_3_I_3_ units that are interconnected by *cyclo*-P_5_ ligands utilizing all five P atoms for coordination (Figure 3f). Three P atoms each form a single η^1^ Cu–P bond to three different Cu_3_I_3_ nodes, while two adjacent P atoms coordinate to a fourth node through two η^1^ Cu–P interactions. Consequently, each *cyclo*-P_5_ ligand connects five metal nodes and therefore acts as a pentatopic linker (Figure 3f). The Cu_3_I_3_ nodes are likewise five-fold-connected and are linked by the *cyclo*-P_5_ ligands to generate an extended 2D CP. Like polymer **4**, the metal nodes in **6** also consist of Cu_6_I_6_ clusters comprising two alternating Cu–I strands arranged opposite to each other and forming five fused Cu_2_I_2_ rhomboidal units (Figure 3g) with short Cu···Cu distances (2.896 to 3.012 Å) [33]. In analogy to **4**, neighboring Cu_6_I_6_ nodes are connected by pairs of *cyclo*-P_5_ ligands that bridge adjacent clusters and propagate the polymer chain. However, in contrast to polymer **4**, the remaining *cyclo*-P_5_ ligands coordinated to each node do not act as terminal ligands but instead establish additional connections between neighboring chains (Figure 3h). Through these interchain linkages, adjacent 1D motifs become crosslinked, ultimately resulting in the formation of an extended 2D CP layer. The P–P bond lengths in polymers **2**–**6** fall within the ranges 2.1108(7)–2.1697(7) Å (**2**), 2.0961(19)–2.1600(19) Å (**3**), 2.096(8)–2.173(5) Å (**4**), 2.094(2)–2.111(3) Å (**5**) and 2.098(3)–2.109(3) Å (**6**), respectively. Thus, all P–P bonds are slightly elongated compared to those in the starting material [Cp″Fe(η^5^-P_5_)] (2.095(3)–2.117(3) Å), which can be attributed to coordination of the *cyclo*-P_5_ ligand to Cu centers. In polymers **2** and **3**, the longest P–P bonds are observed for P atoms involved in η^2^ coordination to Cu centers (P1–P2 in **2** and P2–P3 in **3**; Figure 2a,d), consistent with a partial weakening of the corresponding P–P bonds upon metal coordination. In contrast, polymer **4** does not exhibit η^2^-coordination; nevertheless, one P–P bond associated with the terminal *cyclo*-P_5_ ligand displays a comparable elongation. For polymers **5** and **6**, in which only η^1^-coordination modes are present, the P–P bond lengths are more uniform, and no pronounced elongation is observed. The mean Fe-P bond lengths in polymers **2**–**6** are 2.3751(6) Å (**2**), 2.378(15) Å (**3**), 2.376(3) Å (**4**), 2.391(2) Å (**5**) and 2.375(3) Å (**6**), respectively, and are comparable to that observed for the starting material **1**″ (2.370(3) Å). The Cu–P bond lengths in polymers **2**–**6** span the ranges of 2.1922(6)–2.3666(6) Å for (**2**), 2.2234(16)–2.4304(16) Å for (**3**), 2.203(7)–2.310(7) Å for (**4**), 2.2555(18)–2.299(2) Å for (**5**) and 2.270(2)–2.278(2) Å for (**6**). In polymers **2** and **3**, the longest Cu–P bond lengths are observed for the P atoms involved in η^2^-coordination to Cu centers, whereas η^1^-coordinated P atoms display shorter Cu–P bonds. For polymers **4** and **6**, the Cu–P distances are comparatively uniform, reflecting similar CuI coordination environments and node geometries in these structures. In polymer **5**, four Cu–P bond lengths are comparable to those observed in polymers **4** and **6**, while one slightly shorter Cu–P bond is present.

Crystals of polymers **2**–**6** exhibit limited solubility in CH_2_Cl_2_, THF and CH_3_CN but remain insoluble in nonpolar solvents such as alkanes. Their solution behavior was investigated by multinuclear NMR spectroscopy and electrospray ionization mass spectrometry (ESI-MS). The ^31^P{^1^H} NMR spectra of all polymers recorded in CD_2_Cl_2_/CD_3_CN display single sharp resonances within a narrow chemical shift range of δ = 163.4–165.6 ppm, slightly shifted to that observed for the free ligand **1**″ (δ = 166.4). The presence of a single resonance in each case suggests dynamic behavior involving rapid exchange of Cu–P interactions on the NMR timescale. Importantly, although the signals occur in a similar chemical shift region to that of the free ligand, they are not strictly identical, which is consistent with retention of metal coordination in solution rather than complete depolymerization. The ^1^H NMR spectra of polymers **2**–**6** show signals exclusively attributable to the [Cp″Fe(η^5^-P_5_)] ligand framework. The Cp″ ring proton resonances appear slightly downfield (δ = 4.03–4.20 ppm) relative to the free ligand **1**″ (δ = 3.63–3.65 ppm), consistent with deshielding induced by coordination of the *cyclo*-P_5_ ligand to Cu centers. Positive ESI-MS ion mode measurements performed in CH_3_CN at room temperature reveal a consistent pattern of mono- and multinuclear Cu-containing species across the polymer series. Prominent ions include fragments of the type [(**1**″)_2_Cu_3_X_2_]^+^, [(**1**″)_2_Cu_2_X]^+^, [(**1**″)Cu_2_X]^+^, [(**1**″)CuX]^+^ and [(**1**″)_2_Cu]^+^ (X = Cl, Br, I). The detection of these metal-containing fragments is consistent with Cu–P interactions persisting in solution and that the polymers undergo partial fragmentation into smaller coordination units rather than complete dissociation into the free ligand. Taken together, the combined NMR and ESI-MS data support the presence of persistent Cu–P coordination in solution and suggest that the polymers **2**–**6** undergo partial fragmentation into smaller coordination assemblies rather than complete dissociation into free ligand and copper halide species.

### 2.2. Self-Assembly of [Cp‴Fe(η^5^-P_5_)] (**1‴**) with CuX (X = Cl, Br, I)

**Scheme 2:**
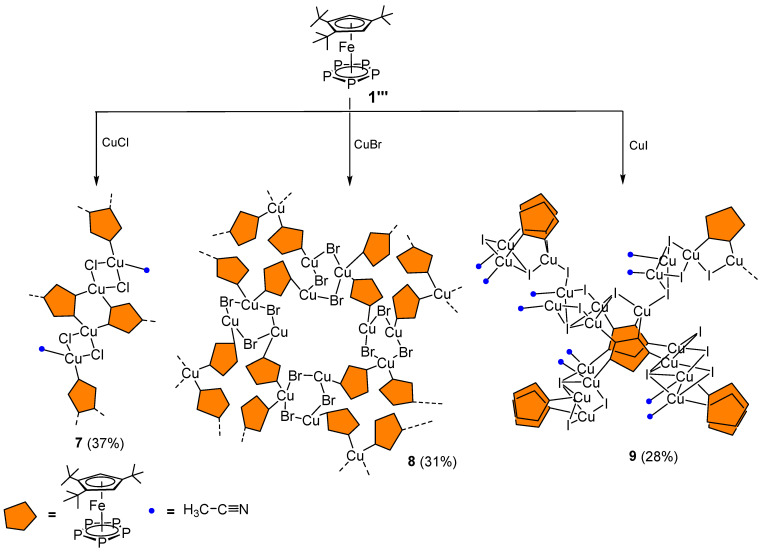
Self-assembly of **1‴** with CuX (X = Cl, Br and I). Schematic presentation of sections of the formed CPs **7**–**9**; isolated yields are given in parenthesis.

**Figure 4:**
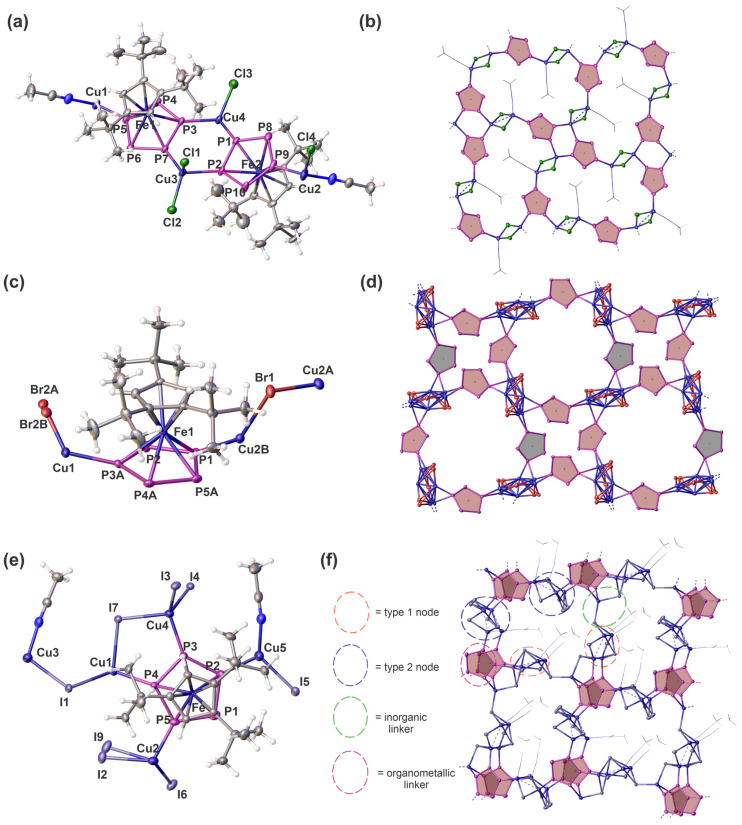
Molecular structures of (**a**) **7**, (**c**) **8** and (**e**) **9**. Sections of the 2D frameworks in (**b**) **7**, (**d**) **8** and (**f**) **9**. Selected bond lengths (Å) and angles (deg) for **7**: P1-P2 2.1000(1), P1-P8 2.1023(1), P2-P10 2.0990(1), P3-P7 2.1066(1), P3-P4 2.0938(1), P7-P6 2.1102(1), Cu3-P7 2.2414(9), Cu3-P2 2.3486(9), Cu4- P3 2.2817(9), Cu4-P1 2.2302(9), Cu1-P5 2.2123(9), Cu2-P9 2.1866(1), P1-Cu4-P3 107.43(3), P7-Cu3-P2 108.27(3). Selected bond lengths (Å) and angles (deg) for **8**: P1-P2 2.1011(1), P1-P5 2.177(7), P2-P3 2.064(7), P3-P4 2.121(6), P4-P5 2.110(5), Cu1-P3 2.245(9), Cu2-P1 2.267(2), Cu2A-Br1-Cu2B 108.20(3), P3A-Cu1-Br2A 105.0(2), P3A-Cu1-Br2B 113.9(2). Selected bond lengths (Å) and angles (deg) for **9**: P2-P1 2.0982(2), P5-P1 2.0861(2), P4-P5 2.1082(2), P4-P5 2.1082(2), P3-P4 2.1025(2), Cu4-P3 2.2596(1), Cu1-P4 2.3552(1), Cu2-P5 2.2508(13), Cu5-P2 2.2485(1), P4-P3-P2 106.83(6), Cu1-I7-Cu4 102.37(2), P4-P3-Cu4 117.18(6), P2-P3-Cu4 135.87(6). Extended polymeric network/connection is shown by the dotted lines.

In a second approach, [Cp‴Fe(η^5^-P_5_)] (**1‴**) was reacted with copper halides CuX (X = Cl, Br, I). In contrast to the behavior observed for **1″**, whose reactivity is sensitive to the stoichiometric ratio of the reactants, the reactions of **1‴** with copper halides displayed no such dependence. In a typical experiment, a solution of **1‴** (one equiv.) in CH_2_Cl_2_ was layered with two equiv. CH_3_CN solutions of CuCl, CuBr or CuI. Within a few days, crystalline products **7**–**9** were obtained, respectively, as brown (**7** and **8**) and red (**9**) crystals in good isolated yields (28–37%, Scheme 2). Compound **7** crystallizes in the monoclinic space group P2_1_/n and forms a 2D CP with the general formula [{Cp**‴**Fe(μ,η^5^:η^1^:η^1^:η^1^-P_5_)}_2_(CuCl)(CuCl)_2_(CuCH_3_CN)(CuCH_3_CN)Cl]_∞_ (**7**) (Scheme 2, Table 1). The asymmetric unit contains two crystallographically independent *cyclo*-P_5_ ligands adopting 1,2,4-coordination mode together with four Cu(I) centers (Figure 4a). A characteristic structural feature is the direct linkage of neighboring *cyclo*-P_5_ rings through pairs of Cu ions coordinated at adjacent 1,2-positions, resulting in the formation of six-membered P–P–Cu–P–P–Cu ring motifs that propagate throughout the lattice (Figure 4a). All Cl ligands act as μ_2_-bridging donors, interconnecting adjacent copper centers and establishing a continuous metal–halide backbone. From a connectivity perspective, the framework can be rationalized in terms of Cu_2_Cl_2_(CH_3_CN) nodes in which each Cu atom adopts a distorted tetrahedral coordination environment (Figure 4b). Despite their similar coordination numbers, two distinct coordination patterns can be distinguished. One group of Cu centers is bound to two P atoms and two μ_2_-Cl ligands, whereas the second group coordinates to one P atom, one N donor from an acetonitrile ligand, and two μ_2_-Cl ligands (Figure 4b). Propagation of these nodes through *cyclo*-P_5_ linkers generates an extended 2D sheet-like framework composed of regularly arranged rectangular meshes. Each mesh is defined by four Cu_2_Cl_2_(CH_3_CN) nodes connected by four *cyclo*-P_5_ ligands. Within this motif, the *cyclo*-P_5_ rings establish two η^1^ Cu–P contacts at the 1,3-positions, while an additional η^1^ interaction links adjacent meshes, thereby sustaining the continuous 2D framework (Figure 4b).

Compound **8** crystallizes in the tetragonal space group I4 and likewise adopts a 2D coordination topology, albeit based on a distinct structural motif (Scheme 2, Table 1). The asymmetric unit comprises one *cyclo*-P_5_ ligand, three Cu(I) centers, and three Br ions. Two of the Cu and all Br ions exhibit crystallographic occupancies of 0.5, as they reside on special positions within the unit cell (Figure 4c). The structure may be described in terms of Cu_3_Br_3_ nodes that are disordered over six Cu and five Br positions per node, preventing an unambiguous assignment of the exact connectivity within the cluster units (Figure 4d). Each *cyclo*-P_5_ linker coordinates adjacent nodes through Cu–P interactions involving the 1,3-positions of the *cyclo*-P_5_ ring, thereby propagating the framework in two dimensions (Figure 4d). A notable feature of the resulting network is the presence of two distinct mesh sizes within the same sheet. The periodic alternation of smaller and larger meshes generates a patterned two-dimensional architecture with pronounced long-range structural regularity (Figure 4d).

Compound **9** crystallizes in the orthorhombic space group *Pnma* and forms a double-layered 2D hybrid CP assembled from a combination of distinct linker and node types (Scheme 2, Table 1). The asymmetric unit contains one *cyclo*-P_5_ ligand coordinated through four of its P atoms, while the remaining P atom remains non-coordinated. In addition, 4.5 Cu(I) centers and 4.5 I ions are distributed over five and eight crystallographic positions, respectively, with occupancies of either 0.5 or 1.0 depending on the individual atom sites (Figure 4e). The structure can be rationalized in terms of the different nodes and linker motifs constituting the 2D framework. Each mesh contains four nodes that can be divided into two distinct types. Type 1 nodes correspond to Cu_4_I_3_(CH_3_CN)_2_ units composed of two five-coordinate Cu(I) centers lacking coordinated acetonitrile ligands and two six-coordinate Cu(I) centers each bearing one coordinated CH_3_CN molecule. Within these nodes, two types of I bridging modes are observed, namely µ_2_-I bridges together with one µ_4_-I ligand capping all Cu centers (Figure 4f). Type 2 nodes are represented by Cu_4_I_4_(CH_3_CN)_2_ units in which all Cu(I) centers are pentacoordinated. Similar to type 1, two different iodide bridging modes occur within these nodes, comprising three µ_2_-I bridges and one µ_4_-I ligand connecting all four Cu centers (Figure 4f). Interestingly, the type 1 nodes are interconnected through two distinct linker species. The first linker type is provided by the *cyclo*-P_5_ ligand of **1‴**, which coordinates via η^1^ Cu–P interactions. The second linker type consists of an inorganic CuI_2_ fragment featuring µ_3_-I bridges. In this unit, each I ion bridges two neighboring Cu centers belonging to adjacent type 1 nodes, such that the complete CuI_2_ fragment acts as an inorganic linker between neighboring nodes (Figure 4f). The Cu center within this linker additionally bridges two P atoms originating from two separate *cyclo*-P_5_ ligands and thereby contributes to closure of the framework meshes. In contrast, the type 2 nodes are interconnected exclusively through *cyclo*-P_5_ linkers, with each node coordinated to four *cyclo*-P_5_ units through individual η^1^ Cu–P interaction. Overall, the connectivity in **9** arises from the cooperative interplay between the different node and linker type, which alternate throughout the lattice to generate the double-layered 2D topology (Figure 4f). Although minor variations in Cu···Cu separations are observed among the individual nodes, these differences do not influence the overall connectivity or dimensionality of the framework. To the best of our knowledge, structurally characterized extended coordination polymers in which metal or metal-cluster nodes are simultaneously interconnected by both organometallic metalloligands (**1‴** in the present case) and purely inorganic linkers (CuI_2_ fragments in the present case) have not been previously reported.

The P–P bond lengths in polymers **7**–**9** lie within the ranges 2.0938(11)–2.1102(12) Å (**7**), 2.034(8)–2.177(7) Å (**8**), and 2.0861(18)–2.1082(16) Å (**9**). These values largely overlap with those observed for the starting material **1‴** (2.1028(5)–2.1137(5) Å). Polymer **7** displays a particularly narrow distribution of P–P distances, consistent with a uniform coordination environment. In contrast, polymer **8** exhibits the widest spread of values, reflecting the presence of crystallographically distinct metalloligand environments within its framework, whereas polymer **9** again shows a more consistent set of P–P distances across the structure. The mean Fe-P bond lengths in polymers **7**–**9** are 2.388 Å (**7**), 2.378 Å (**8**) and 2.395 Å (**9**), which remain very close to that of **1‴** (2.378 Å). The Cu–P bond lengths are in the ranges 2.1866(10)–2.3486(9) Å (**7**), 2.215(2)–2.536(11) Å (**8**) and 2.2485(13)–2.3553(13) Å (**9**). 

Crystals of polymers **7**–**9** exhibit improved solubility in CH_2_Cl_2_ and THF compared to derivatives **2**–**6**; however, they remain insoluble in nonpolar solvents such as alkanes. The ^1^H NMR spectra of all three compounds in solution display resonances associated with the Cp‴ ligand with the CpH protons appearing slightly downfield shifted (δ ≈ 4.1 ppm) relative to the corresponding signal in **1‴** (δ = 3.95 ppm). The ^31^P{^1^H} NMR spectra of polymers **7**–**9** each show a single resonance at δ = 166.8 ppm (**7**), 162.4 ppm (**8**) and 165.9 ppm (**9**), respectively, close to that observed for the starting material **1‴** (δ = 165.2 ppm). In the positive ESI-MS ion mode measurements performed in CH_3_CN at room temperature, some or all the following peaks attributable to [(**1‴**)Cu(CH_3_CN)]^+^, [(**1‴**)_2_Cu]^+^, [(**1‴**)_2_Cu_2_X]^+^ (X = Cl, Br, I) as well as higher nuclear aggregates containing multiple Cu centers were detected. The detection of such fragments demonstrates that the polymers undergo only partial fragmentation in solution.

Gas-phase DFT calculations (B3LYP/def2-TZVP) [34,35,36,37,38] on the [Cp^R^Fe(η^5^-P_5_)] (R = Cp*, Cp″ and Cp**‴**) moieties indicate broadly comparable electronic structures across Cp*, Cp″ and Cp**‴**, despite the loss of HOMO/LUMO degeneracy upon *^t^*Bu substitution and only modest changes in frontier orbital energies (for further information see ESI). These results suggest that the markedly different CuX self-assembly behavior is unlikely to arise from electronic effects alone and is instead most plausibly attributed to increased steric demand imposed by the tert-butyl substituted Cp^R^ ligands.

## 3. Materials and Methods

### 3.1. General Information

All manipulations were carried out under a dry argon or dinitrogen atmosphere using glovebox or standard Schlenk techniques. The solvents were taken from a solvent purification system of the MB-SPS-800 type from the company MBRAUN (Garching, Germany) and degassed by standard procedures. The starting materials [Cp**″**Fe(η^5^-P_5_)] (**1″**) [32] and [Cp‴Fe(η^5^-P_5_)] (**1‴**) [32] were prepared according to procedures outlined in the literature. Compounds CuCl, CuBr and CuI were purchased from Merck (Darmstadt, Germany) and used without further purification. The NMR spectra were recorded on a Bruker Avance 400 using TopSpin version 5.0.0 (Billerica, MA, USA, ^1^H, 400.132 MHz; ^31^P, 161.975 MHz) referenced to external SiMe_4_ (^1^H) or H_3_PO_4_ (^31^P), respectively. HRMS (ESI) spectra were recorded on an Agilent 6200 Series LC-QTOF mass spectrometer (Agilent Technologies, Santa Clara, CA, USA) in positive-ion mode. Elemental analyses (C, H, N) were determined on a Vario EL III instrument (Elementar Analysensysteme GmbH, Donaustraße 7, 63452 Hanau, Germany).

### 3.2. Synthesis and Characterization of Polymers **2**–**9**

#### 3.2.1. Synthesis and Characterization of Polymer **2**

A CH_2_Cl_2_ solution of [Cp**″**Fe(η^5^-P_5_)] (**1″**, 40 mg, 0.1 mmol) was carefully layered with a 1:1 mixture of CH_2_Cl_2_ and CH_3_CN (4 mL), which was subsequently overlaid with a CH_3_CN solution of CuCl (20 mg, 0.2 mmol). After complete diffusion and homogenization of the phases, no crystalline material was initially observed. The reaction mixture, however, turned brown, in contrast to the green color of the starting material pentaphosphaferrocene **1″**. The crude mixture was filtered and layered with a three-fold volume of *n*-hexane, affording red crystals of **2**. Yield: 42 mg (48%). ^1^H NMR (400 MHz, CD_3_CN/CD_2_Cl_2_): δ/ppm = 1.18 (s, Me, 18H), 4.05 (s, CpH, 1H), 4.16 (s, CpH, 2H). ^31^P{^1^H} NMR (162.04 MHz, CD_3_CN/CD_2_Cl_2_): δ/ppm = 165.27 (s). EI-MS (pos.): *m*/*z* (%) = 1036.7 (0.2) [(Cp″FeP_5_)_2_Cu_3_Cl_2_]^+^, 936.8 (0.3) [(Cp″FeP_5_)_2_Cu_2_Cl]^+^, 838.9 (1.5) [(Cp″FeP_5_)_2_Cu]^+^, 589.8 (0.7) [(Cp″FeP_5_)Cu_2_Cl(CH_3_CN)]^+^, 491.9 (2.6) [(Cp″FeP_5_)Cu(CH_3_CN)]^+^, 145.0 (100) [Cu(CH_3_CN)_2_]^+^. Elemental analysis, calcd. for C_17_H_27_Cl_4_Cu_4_FeN_2_P_5_ (866.12 g/mol): C, 23.57; H, 3.14; N, 3.23. Found C: 23.22%, H: 3.19%, N: 2.90%.

#### 3.2.2. Synthesis and Characterization of Polymer **3**

Similar reaction procedure to that of **2**, involving 40 mg of **1″** and 29 mg CuBr. Polymer **3** was obtained as brown crystals. Yield: 40 mg (45%). ^1^H NMR (400 MHz, CD_3_CN/CD_2_Cl_2_): δ/ppm = 1.18 (s, Me, 18H), 4.07 (s, CpH, 1H), 4.20 (s, CpH, 2H). ^31^P{^1^H} NMR (162.04 MHz, CD_3_CN/CD_2_Cl_2_): δ/ppm = 163.91 (s). EI-MS (pos.): *m*/*z* (%) = 1126.6 (0.5) [(Cp″FeP_5_)_2_Cu_3_Br_2_]^+^, 980.7 (1.4) [(Cp″FeP_5_)_2_Cu_2_Br]^+^, 838.9 (7.9) [(Cp″FeP_5_)_2_Cu]^+^, 633.8 (5.1) [(Cp″FeP_5_)Cu_2_Br(CH_3_CN)]^+^, 491.9 (7.4) [(Cp″FeP_5_)Cu(CH_3_CN)]^+^, 145.0 (100) [Cu(CH_3_CN)_2_]^+^. Elemental analysis, calcd. for C_17_H_27_Br_3_Cu_3_FeN_2_P_5_ (900.48 g/mol): C, 22.67; H, 3.02; N, 3.11. Found C: 23.31%, H: 3.33%, N: 3.26%.

#### 3.2.3. Synthesis and Characterization of Polymer **4**

Similar reaction procedure to that of **2**; however, it involved a 1:1 ratio of **1″** (40 mg, 0.1 mmol) to CuI (19 mg, 0.1 mmol). Polymer **4** was obtained as yellow crystals. Yield: 22 mg (33%). ^1^H NMR (400 MHz, CD_3_CN/CD_2_Cl_2_): δ/ppm = 1.18 (s, Me, 18H), 4.03 (s, CpH, 1H), 4.14 (s, CpH, 2H). ^31^P{^1^H} NMR (162.04 MHz, CD_3_CN/CD_2_Cl_2_): δ/ppm = 165.62 (s). EI-MS (pos.): *m*/*z* (%) = 1220.5 (0.3) [(Cp″FeP_5_)_2_Cu_3_I_2_]^+^, 1028.7 (0.6) [(Cp″FeP_5_)_2_Cu_2_I]^+^, 838.9 (2.9) [(Cp″FeP_5_)_2_Cu]^+^, 681.8 (0.8) [(Cp″FeP_5_)Cu_2_I(CH_3_CN)]^+^, 491.9 (2.4) [(Cp″FeP_5_)Cu(CH_3_CN)]^+^, 145.0 (100) [Cu(CH_3_CN)_2_]^+^. Elemental analysis, calcd. for C_26_H_42_Cu_3_Fe_2_I_3_P_10_ (1347.35 g/mol): C, 19.12; H, 2.59. Found C: 19.33%, H: 2.79%.

#### 3.2.4. Synthesis and Characterization of Polymers **5** and **6**

Similar reaction procedure to that of **4**, involving however a 1:2 ratio of **1″** (40 mg, 0.1 mmol) to CuI (38 mg, 0.2 mmol). Crystals of both **5** (yellow plates) and **6** (red plates) are obtained from the reaction mixture which can be manually separated from one another. ^1^H NMR (400 MHz, CD_3_CN/CD_2_Cl_2_, **5**): δ/ppm = δ/ppm = 1.17 (s, Me, 18H), 4.05 (s, CpH, 1H), 4.18 (s, CpH, 2H). ^31^P{^1^H} NMR (162.04 MHz, CD_3_CN/CD_2_Cl_2_, **5**): δ/ppm = 163.35 (s). ^1^H NMR (400 MHz, CD_3_CN/CD_2_Cl_2_, **6**): δ/ppm = 1.18 (s, Me, 18H), 4.05 (s, CpH, 1H), 4.16 (s, CpH, 2H). ^31^P{^1^H} NMR (162.04 MHz, CD_3_CN/CD_2_Cl_2_, **6**): δ/ppm = 163.41 (s). Yield (**5**): 10 mg (18%); Yield (**6**): 20 mg (21%). EI-MS (**5**, pos.): *m*/*z* (%) = 1220.5 (2.0) [(Cp″FeP_5_)_2_Cu_3_I_2_]^+^, 1028.7 (5.7) [(Cp″FeP_5_)_2_Cu_2_I]^+^, 838.9 (28.8) [(Cp″FeP_5_)_2_Cu]^+^, 681.8 (2.1) [(Cp″FeP_5_)Cu_2_I(CH_3_CN)]^+^, 491.9 (15.7) [(Cp″FeP_5_)Cu(CH_3_CN)]^+^, 145.0 (100) [Cu(CH_3_CN)_2_]^+^. EI-MS (**6**, pos.): *m*/*z* (%) = 1220.5 (1.8) [(Cp″FeP_5_)_2_Cu_3_I_2_]^+^, 1028.7 (5.0) [(Cp″FeP_5_)_2_Cu_2_I]^+^, 838.9 (28.0) [(Cp″FeP_5_)_2_Cu]^+^, 681.8 (1.9) [(Cp″FeP_5_)Cu_2_I(CH_3_CN)]^+^, 491.9 (13.9) [(Cp″FeP_5_)Cu(CH_3_CN)]^+^, 145.0 (100) [Cu(CH_3_CN)_2_]^+^. Elemental analysis, calcd. for C_13_H_21_Cu_3_FeI_3_P_5_ (**5**, 959.35 g/mol): C, 16.27; H, 2.21. Found C: 16.65%, H: 2.41%. calcd. for C_39_H_63_Cu_6_Fe_3_I_6_P_15_ (**6**, 2306.69 g/mol): C, 23.18; H, 3.14. Found C: 23.17%, H: 3.28%.

#### 3.2.5. Synthesis and Characterization of Polymer **7**

A CH_2_Cl_2_ solution of [Cp**‴**FeP_5_] (**1‴**, 44 mg, 0.1 mmol) was carefully layered with a CH_3_CN solution of CuCl (20 mg, 0.2 mmol). After complete diffusion and homogenization of the phases, no crystalline material was observed. The crude mixture was filtered and layered with a three-fold volume of *n*-hexane, from which brown crystals of **7** were obtained. Yield: 25 mg (37%). ^1^H NMR (400 MHz, CD_3_CN/CD_2_Cl_2_): δ/ppm = 1.13 (s, Me, 18H), 1.20 (s, Me, 9H), 3.96 (s, CpH, 1H), 4.11 (s, CpH, 1H). ^31^P{^1^H} NMR (162.04 MHz, CD_3_CN/CD_2_Cl_2_): δ/ppm = 166.0 (s). EI-MS (pos.): *m*/*z* (%) = 951.0 (0.3) [(Cp‴FeP_5_)_2_Cu]^+^, 645.9 (0.4) [(Cp**‴**FeP_5_)Cu_2_Cl(CH_3_CN)]^+^, 548.0 (1.8) [(Cp‴FeP_5_)Cu(CH_3_CN)]^+^, 145.0 (100) [Cu(CH_3_CN)_2_]^+^. Elemental analysis, calcd. for C_38_H_64_Cl_4_Cu_4_Fe_2_N_2_P_10_ (1366.31 g/mol): C, 33.40; H, 4.72, N, 2.05. Found C: 34.09%, H: 4.76%, N: 1.68%.

#### 3.2.6. Synthesis and Characterization of Polymer **8**

Similar reaction procedure to that of **7**, involving 44 mg of **1‴** and 29 mg CuBr. Polymer **8** was obtained as dark brown crystals. Yield: 20 mg (31%).^1^H NMR (400 MHz, CD_3_CN/CD_2_Cl_2_): δ/ppm = 1.19 (s, Me, 9H), 1.33 (s, Me, 18H), 4.12 (s, CpH, 2H). ^31^P{^1^H} NMR (162.04 MHz, CD_3_CN/CD_2_Cl_2_): δ/ppm = 162.37 (s). EI-MS (pos.): *m*/*z* (%) = 1236.7 (2.5) [(Cp‴FeP_5_)_2_Cu_3_Br_2_]^+^, 1094.8 (5.7) [(Cp‴FeP_5_)_2_Cu_2_Br]^+^, 951.0 (6.1) [(Cp‴FeP_5_)_2_Cu]^+^, 689.8 (1.1) [(Cp‴FeP_5_)Cu_2_Br(CH_3_CN)]^+^, 548.0 (6.2) [(Cp‴FeP_5_)Cu(CH_3_CN)]^+^, 145.0 (100) [Cu(CH_3_CN)_2_]^+^. Elemental analysis, calcd. for C_34_H_58_Br_3_Cu_3_Fe_2_P_10_ (1318.55 g/mol): C, 30.97; H, 4.43. Found C: 30.94%, H: 4.41%.

#### 3.2.7. Synthesis and Characterization of Polymer **9**

Similar reaction procedure to that of **7**, involving 44 mg of **1‴** and 38 mg CuI. Polymer **9** was obtained as red crystals. Yield: 21 mg (28%). ^1^H NMR (400 MHz, CD_3_CN/CD_2_Cl_2_): δ/ppm = 1.19 (s, Me, 9H), 1.33 (s, Me, 18H), 4.17 (s, CpH, 2H). ^31^P{^1^H} NMR (162.04 MHz, CD_3_CN/CD_2_Cl_2_): δ/ppm = 165.88 (s). EI-MS (pos.): *m*/*z* (%) = 1332.7 (1.3) [(Cp‴FeP_5_)_2_Cu_3_I_2_]^+^, 1142.8 (2.0) [(Cp‴FeP_5_)_2_Cu_2_I]^+^, 951.0 (18.7) [(Cp‴FeP_5_)_2_Cu]^+^, 737.8 (1.1) [(Cp‴FeP_5_)Cu_2_I(CH_3_CN)]^+^, 548.0 (21.4) [(Cp‴FeP_5_)Cu(CH_3_CN)]^+^, 144.5 (100) [Cu(CH_3_CN)_2_]^+^. Elemental analysis, calcd. for C_42_H_70_Cu_9_Fe_2_I_9_N_4_P_10_ (2766.47 g/mol): C, 18.23; H, 2.55, N, 2.03. Found C: 18.10%, H: 2.59%, N: 1.89%.

### 3.3. Crystallography

The crystallographic data for polymers **2**, **8** and **9** was collected on a Rigaku XtaLAB Synergy R diffractometer (Akishima, Japan) equipped with a HyPix-Arc 150 detector using Cu-K_α_ radiation (λ = 1.54184 Å). Data for polymers **3**, **4**, and **6** was collected on a Agilent Technologies SuperNova diffractometer (Agilent Technologies XRD Products, 10 Mead Road, Yarnton, Oxfordshire, OX5 1QU, UK) equipped with a TitanS2 detector using Cu-K_α_ radiation (λ = 1.54184 Å). Polymer **5** was measured on a Agilent Technologies SuperNova diffractometer (Agilent Technologies XRD Products, 10 Mead Road, Yarnton, Oxfordshire, OX5 1QU, UK) equipped with an Eos CCD detector using Mo-K_α_ radiation (λ = 0.71073 Å), while polymer **7** was measured on a Agilent Technologies SuperNova Dualflex diffractometer (Agilent Technologies XRD Products, 10 Mead Road, Yarnton, Oxfordshire, OX5 1QU, UK) equipped with a TitanS2 detector using Cu-K_α_ radiation (λ = 1.54184 Å). Data collection, cell refinement, and data reduction were performed using the CrysAlisPro software (versions 1.171.41.54a, 1.171.41.90a, and 1.171.41.123a) [39]. Structures were solved with Olex2 (1.5-alpha) [40] using ShelXT-2018/2 [41] and a least-square refinement on *F*^2^ was carried out with ShelXL-2018/3 [42]. Absorption corrections were applied using analytical, numerical, Gaussian integration, or multi-scan methods, with empirical corrections based on spherical harmonics implemented in the SCALE3 ABSPACK scaling algorithm where appropriate. Hydrogen atoms were placed in calculated positions and refined using riding models. Crystallographic drawings and publication materials were prepared using Olex2. CCDC reference numbers 2558806-2558813 contain supplementary crystallographic data which is deposited in the Cambridge Crystallographic Data Centre.

### 3.4. Computational Details

Gas phase geometry optimizations of the cationic fragments (the calculations did not account for the anionic part) were carried out using the Gaussian 09 (rev. E.01) series of the program [43] with the B3LYP functional [34] in conjunction with def2-TZVP [38] basis set. Note that all geometry optimizations were performed at the B3LYP/TZVP level of theory without inclusion of an empirical dispersion correction. This level of theory was adopted consistently throughout the study to understand the qualitative comparison of the electronic structure. The optimized geometries were characterized as true minima via analytical frequency calculations (zero negative eigenvalues of the Hessian).

## 4. Conclusions

In conclusion we have shown that the self-assembly of [Cp″Fe(η^5^-P_5_)] (**1″**) and [Cp**‴**Fe(η^5^-P_5_)] (**1‴**) with copper halides affords a diverse family of coordination polymers exhibiting a wide range of nuclearities, connectivities, and dimensionalities. These architectures are constructed from copper halide cluster nodes interconnected by *cyclo*-P_5_-based organometallic linkers, underscoring the pronounced structural flexibility of the *cyclo*-P_5_ ligand and its ability to adopt multiple coordination modes in extended frameworks. In contrast to previously reported pentaphosphaferrocene derivatives, which tend to form discrete spherical aggregates upon reaction with copper halides, the present systems consistently assemble into extended coordination polymer networks. This distinct behavior probably coincides with the absence of idealized five-fold symmetry in [Cp″Fe(η^5^-P_5_)] and [Cp‴Fe(η^5^-P_5_)] compared to earlier systems and highlights the importance of ligand symmetry and substitution pattern in directing the self-assembly outcome. Although this class of polymers remains scares, a unique 2D polymer was obtained from the reaction of **1‴** with CuI featuring mixed nodes and linker types. Thus, Cu_4_I_4_ and Cu_4_I_3_ cluster nodes are found to be interconnected by organometallic *cyclo*-P_5_ ligands and purely inorganic Cu–I fragments. This structural motif expands the design principles for coordination polymers based on organometallic ligands and illustrates the potential of pentaphosphaferrocene derivatives as versatile building blocks for complex hybrid inorganic–organometallic networks.

## Figures and Tables

**Table 1:** General information about polymers **2**–**9**.

Polymer	Cu-Halide Node	*cyclo*-P_5_ Coordination Mode	Polymer Dimensionality
**2**	Cu_4_Cl_4_	mixed η^1^, η^2^, and μ_2_-η^1^:η^1^-coordination of P atoms	1D (double-stranded)
**3**	Cu_6_Br_6_	mixed η^1^, η^2^, and μ_2_-η^1^:η^1^-coordination of P atoms	1D
**4**	Cu_6_I_6_	(i) terminal P_5_: η^1^:coordination (ii) bridging P_5_: η^1^:η^1^:η^1^-coordination	1D
**5**	Cu_3_I_3_	P_5_ coordinates through all P atoms via η^1^ Cu–P bonds	2D
**6**	Cu_6_I_6_	P_5_ linkers coordinate via 3× η^1^ Cu–P bonds	2D
**7**	Cu_2_CI_2_	P_5_ linkers coordinate via 3× η^1^ Cu–P bonds	2D
**8**	Cu_3_Br_3_	P_5_ linkers coordinate via 1,3-coordination mode	2D
**9**	Cu_4_I_4_ and Cu_4_I_3_	P_5_ linkers coordinate via 4× η^1^ Cu–P bonds	2D (double-layered)

## Data Availability

NMR spectra, MS spectra, DFT details and crystal data are given in the Supporting Information. Crystallographic data for compounds **2**–**9** have been deposited with the Cambridge Crystallographic Data Center (CCDC 2558806-2558813).

## Supplementary Materials

The following supporting information can be downloaded at: https://www.mdpi.com/article/10.3390/molecules31142507/s1.
