# Supplementary material for: Self-Assembly of Tert-Butyl-Substituted Pentaphosphaferrocenes with Copper Halides: Selective Formation of One- and Two-Dimensional Coordination Polymers
Source: Molecules. 2026 Jul 17;31(14):2507. doi: 10.3390/molecules31142507 (PMC13415053; doi:10.3390/molecules31142507)
Supplement: Supplementary file 1 [file molecules-31-02507-s001.zip › molecules-4409851-supplementary.pdf]

Supporting Information

Self-Assembly of tert-Butyl-Substituted Pentaphosphaferrocenes with Copper Halides: Selective Formation of One- and Two-Dimensional Coordination Polymers

Bijan Mondal, Mehdi Elsayed Moussa, Michael Seidl and Manfred Scheer*

Department of Inorganic Chemistry, University of Regensburg, GER-93040 Regensburg (Germany);
mondal.bijan@gmail.com (B. M.); mehdi.elsayed-mousa@chemie.uni-regensburg.de (M.E.M.);
Michael.Seidl@uibk.ac.at (M.S.)
* Correspondence: manfred.scheer@ur.de; Tel.: +49-(0)941-943-4440; Fax: +49-(0)941-943-4439

Table of Contents:

1. Crystallographic Data……S2
2. NMR Spectra……S6
3. Mass Spectra……S12
4. Computational Details……S15
5. References……S22

1. Crystallographic Details:

The crystallographic data for polymers **2**, **8** and **9** was collected on a Rigaku XtaLAB Synergy R diffractometer equipped with a HyPix-Arc 150 detector using Cu-K_α radiation (λ = 1.54184 Å). Data for polymers **3**, **4**, and **6** was collected on a GV1000 diffractometer equipped with a TitanS2 detector using Cu-K_α radiation (λ = 1.54184 Å). Polymer **5** was measured on a SuperNova diffractometer equipped with an Eos CCD detector using Mo-K_α radiation (λ = 0.71073 Å), while polymer **7** was measured on a SuperNova Dualflex diffractometer equipped with a TitanS2 detector using Cu-K_α radiation (λ = 1.54184 Å). Data collection, cell refinement, and data reduction were performed using the CrysAlisPro software package.[1] Structures were solved with Olex2 (1.5-alpha)[2] using ShelXT[3] and a least-square refinement on F^2 was carried out with ShelXL.[4] Absorption corrections were applied using analytical, numerical, Gaussian integration, or multi-scan methods, with empirical corrections based on spherical harmonics implemented in the SCALE3 ABSPACK scaling algorithm where appropriate. Hydrogen atoms were placed in calculated positions and refined using riding models. Crystallographic drawings and publication materials were prepared using Olex2. CCDC reference numbers 2558806-2558813 contains supplementary crystallographic data and is deposited in Cambridge Crystallographic Data Centre.

Table S1: Selected parameters for the single crystal X-ray diffraction experiments of polymers **2**-**3**.

Compound	2	3	4
CCDC-number	2558806	2558807	2558808
Formula	$C_{17}H_{27}Cl_4Cu_4FeN_2P_5$	$C_{18.5}H_{29.35}Br_3Cl_{0.40}Cu_3FeN_{2.65}P_5$	$C_{26.55}H_{43.10}Cl_{1.10}Cu_3Fe_2I_3P_{10}$
Formula Weight	866.06	944.12	1394.02
Temperature/K	100.01(10)	122.96(12)	123.01(10)
Crystal System	monoclinic	triclinic	triclinic
Space Group	$P2_1/n$	$P\bar{1}$	$P\bar{1}$
a/Å	15.15230(10)	9.9099(5)	10.0746(2)
b/Å	9.69160(10)	13.0583(8)	15.3182(3)
c/Å	20.16410(10)	13.0864(8)	15.9802(4)
α/°	90	74.144(5)	99.112(2)
β/°	105.7210(10)	81.181(5)	103.253(2)
γ/°	90	73.546(5)	102.227(2)
Volume/Å^3	2850.33(4)	1557.02(17)	2289.84(9)
Z	4	2	2
$D_{calc.}$ / g · cm^{-3}	2.018	2.014	2.022
μ/mm^{-1}	13.491	13.339	26.429
F(000)	1712	917	1338
Size/mm^3	0.19×0.11×0.05	0.128×0.059×0.025	0.156 × 0.08 × 0.027
Radiation type	CuK_α	CuK_α	CuK_α
Wavelength/Å	1.54184	1.54184	1.54184
2Θ range for data collection/°	3.260 to 73.180	3.523 to 73.759	7.428 to 146.992
Index ranges	-18 ≤ h ≤ 12,<br>-12 ≤ k ≤ 11,<br>-24 ≤ l ≤ 24	-11 ≤ h ≤ 12,<br>-16 ≤ k ≤ 15,<br>-16 ≤ l ≤ 16	-12 ≤ h ≤ 12,<br>-18 ≤ k ≤ 17,<br>-19 ≤ l ≤ 19
Refl. collected	26975	16937	25678
Independent Refl.	5582	6076	8963
R_{int}	0.0177	0.0544	0.0642
R_{sigma}	0.0130	0.0532	0.0640
Data/restraints/ parameters	5582/24/316	6076/98/363	8963/48/578
Goodness-of-fit on F^2	1.092	1.067	1.051
wR_2 (all data)	0.0489	0.1457	0.1410
wR_2	0.0487	0.1400	0.1345
R_1 (all data)	0.0215	0.0624	0.0599
R_1	0.0209	0.0548	0.0521
Largest Peak	1.320	1.998	2.313
Deepest Hole	-0.471	–1.091	-1.338

Table S2: Selected parameters for the single crystal X-ray diffraction experiments of polymers **5**-**7**.

**Compound**	**5**	**6**	**7**
CCDC-number	2558809	2558810	2558811
Formula	$C_{13}H_{21}Cu_3FeI_3P_5$	$C_{40.63}H_{66.26}Cl_{3.26}Cu_6Fe_3I_6P_{15}$	$C_{38}H_{64}Cl_4Cu_4Fe_2N_2P_{10}$
Formula Weight	959.32	2445.06	1366.27
Temperature/K	123.00(10)	123.00(10)	123.01(10)
Crystal System	orthorhombic	triclinic	monoclinic
Space Group	$P2_12_12_1$	$P\bar{1}$	$P2_1/n$
a/Å	9.6591(2)	10.1227(3)	15.1831(2)
b/Å	11.4141(2)	14.5644(5)	16.5657(2)
c/Å	22.0307(4)	26.5275(7)	26.4619(3)
α/°	90	101.391(2)	90
β/°	90	96.540(2)	91.1900(10)
γ/°	90	101.929(2)	90
Volume/Å^3	2428.88(8)	3702.0(2)	6654.23(14)
Z	4	2	4
$D_{calc.}$ / g · cm^{-3}	2.623	2.193	1.364
μ/mm^{-1}	7.317	30.430	8.743
F(000)	1784	2321	2768
Size/mm^3	0.12×0.05×0.04	0.14×0.06×0.02	0.3 × 0.12 × 0.05
Radiation type	MoK_{α}	CuK_{α}	CuK_{α}
Wavelength/Å	0.71073	1.54184	1.54184
2Θ range for data collection/°	2.913 to 31.120	6.888 to 148.644	7.9 to 133.8
Index ranges	-11 ≤ h ≤ 13, -15 ≤ k ≤ 16, -28 ≤ l ≤ 29	-11 ≤ h ≤ 12, -17 ≤ k ≤ 16, -32 ≤ l ≤ 26	-18 ≤ h ≤ 17, -19 ≤ k ≤ 19, -31 ≤ l ≤ 31
Refl. collected	13931	24261	59824
Independent Refl.	6820	14237	11713
R_{int}	0.0268	0.0599	0.0827
R_{sigma}	0.0398	0.0734	0.0463
Data/restraints/ parameters	6820/24/233	14237/207/794	11713/72/592
Goodness-of-fit on F^2	1.079	1.029	1.034
wR_2 (all data)	0.0785	0.1575	0.1303
wR_2	0.0768	0.1463	0.1254
R_1 (all data)	0.0358	0.0688	0.0509
R_1	0.0326	0.0570	0.0468
Largest Peak	2.956	1.775	0.960
Deepest Hole	-1.025	–2.026	-0.613

Table S3: Selected parameters for the single crystal X-ray diffraction experiments of polymers **8**,**9**.

**Compound**	**8**	**9**
CCDC-number	2558812	2558813
Formula	$C_{34}H_{58}Br_3Cu_3Fe_2P_{10}$	$C_{21}H_{35}Cu_{4.50}FeI_{4.50}N_2P_5$
Formula Weight	1318.55	1383.19
Temperature/K	123.00(10)	123.00(10)
Crystal System	tetragonal	orthorhombic
Space Group	I4	*Pnma*
a/Å	13.74150(10)	16.41080(10)
b/Å	13.74150(10)	27.7530(2)
c/Å	25.5073(2)	16.27050(10)
α/°	90	90
β/°	90	90
γ/°	90	90
Volume/Å^3	4816.51(8)	7410.38(8)
Z	4	8
$D_{calc.}$ / g · cm^{-3}	1.818	2.480
μ/mm^{-1}	12.433	37.506
F(000)	2624	5160
Size/mm^3	0.09×0.07×0.04	0.064×0.055×0.01
Radiation type	$CuK_α$	$CuK_α$
Wavelength/Å	1.54184	1.54184
2Θ range for data collection/°	6.93 to 139.97	6.3 to 136.50
Index ranges	-16 ≤ h ≤ 14,<br>-16 ≤ k ≤ 16,<br>-27 ≤ l ≤ 31	-19 ≤ h ≤ 14,<br>-33 ≤ k ≤ 33,<br>-19 ≤ l ≤ 19
Refl. collected	12672	73088
Independent Refl.	4384	6936
R_{int}	0.0151	0.0471
R_{sigma}	0.0149	0.0184
Data/restraints/ parameters	4384/0/296	6936/10/370
Goodness-of-fit on F^2	1.037	1.014
wR_2 (all data)	0.0383	0.0714
wR_2	0.0382	0.0706
R_1 (all data)	0.0156	0.0282
R_1	0.0153	0.0267
Largest Peak	0.199	1.327
Deepest Hole	-0.165	–0.921

2. NMR Data:

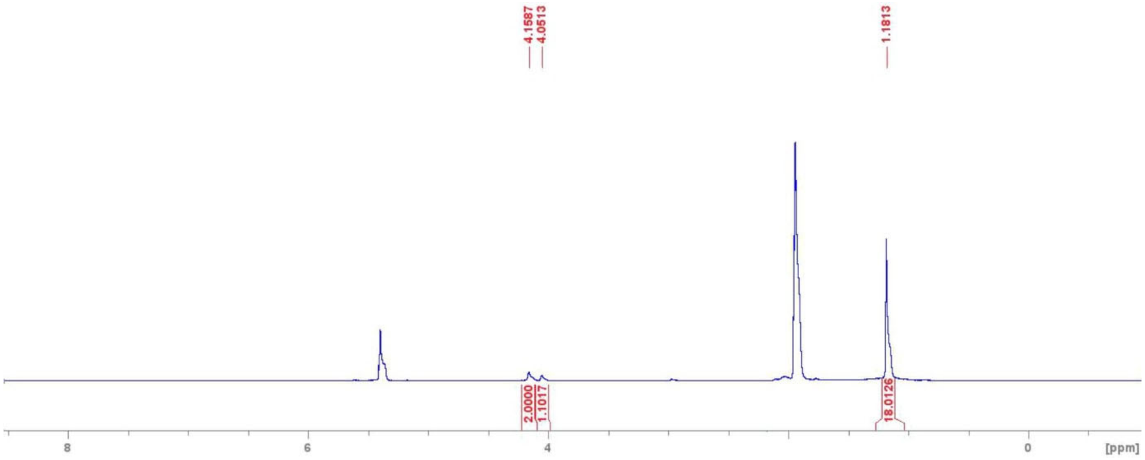

Figure S1 ^{1}H NMR (400.3 MHz, CD_2Cl_2/CD_3CN) spectrum of polymer **2**.

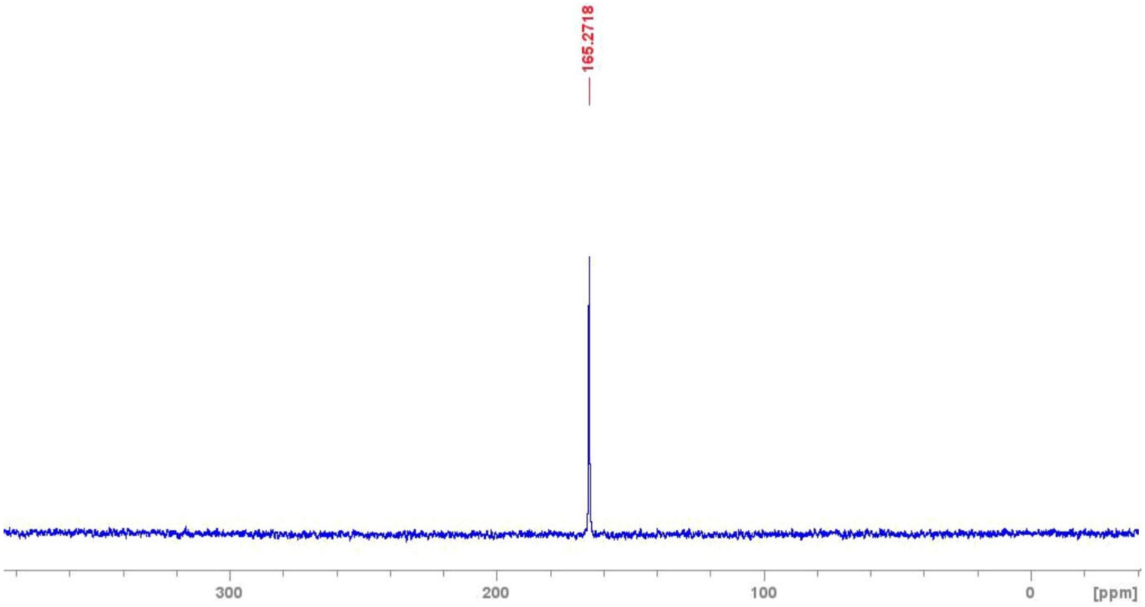

Figure S2 $^{31}P\{^{1}H\}$ NMR (162.0 MHz, CD_2Cl_2/CD_3CN) spectrum of polymer **2**.

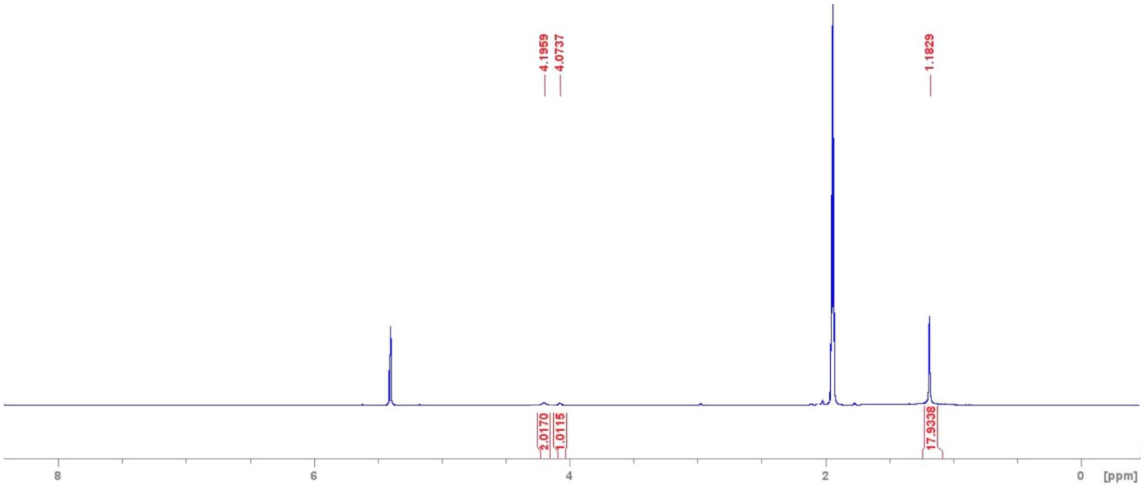

Figure S3 ^{1}H NMR (400.3 MHz, CD_2Cl_2/CD_3CN) spectrum of polymer **3**.

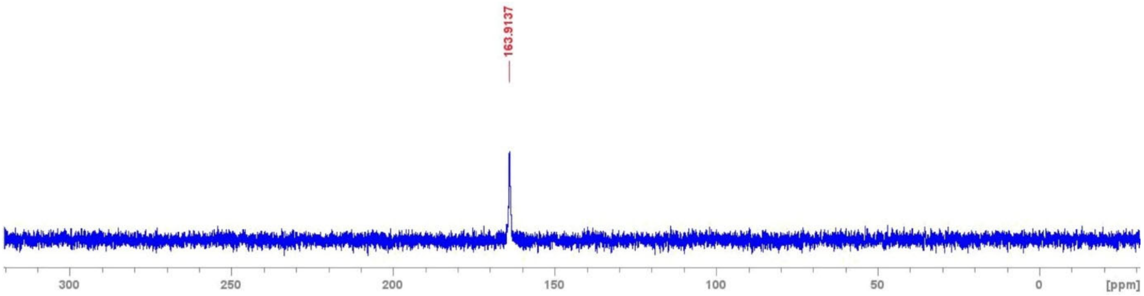

Figure S4 $^{31}P\{^{1}H\}$ NMR (162.0 MHz, CD_2Cl_2/CD_3CN) spectrum of polymer **3**.

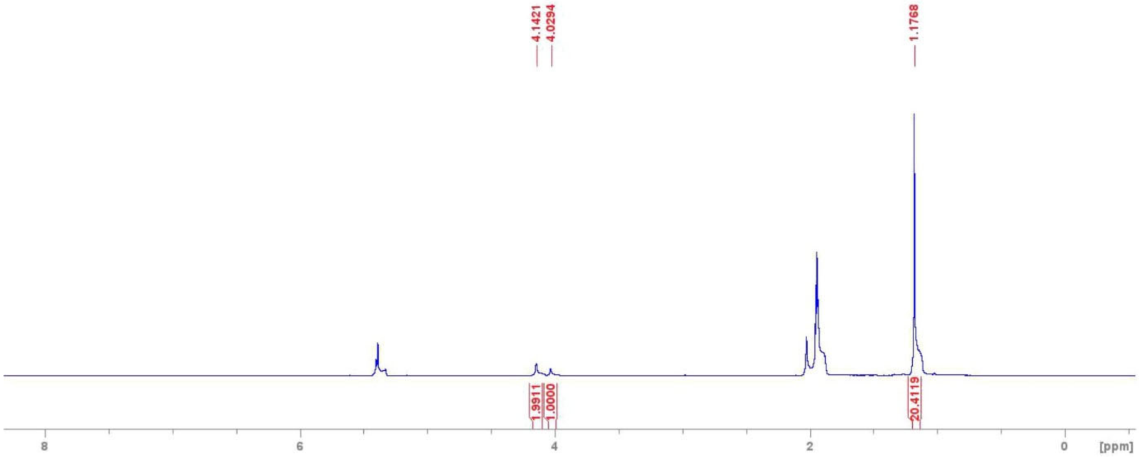

Figure S5 ^{1}H NMR (400.3 MHz, CD_2Cl_2/CD_3CN) spectrum of polymer **4**.

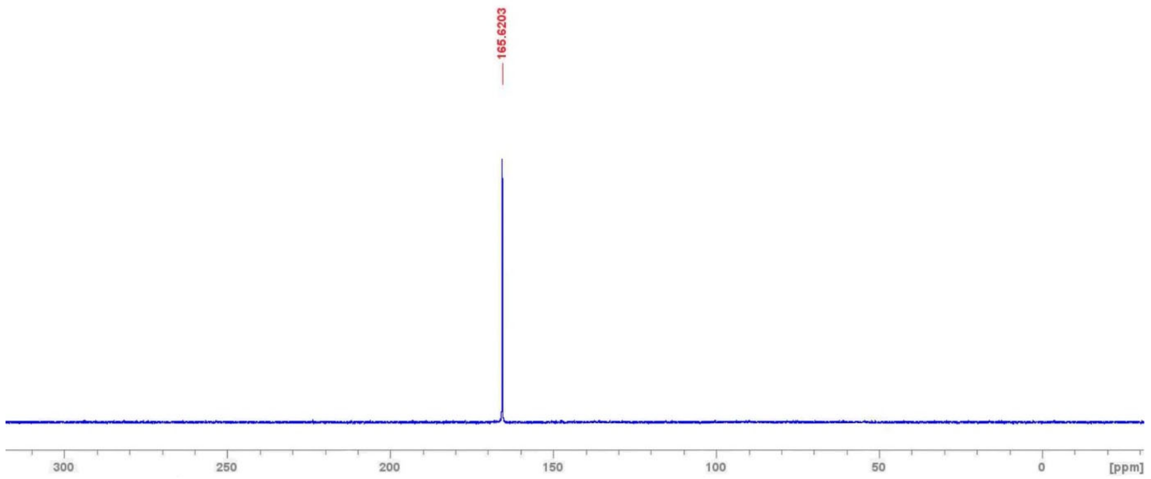

Figure S6 $^{31}P\{^{1}H\}$ NMR (162.0 MHz, CD_2Cl_2/CD_3CN) spectrum of polymer **4**.

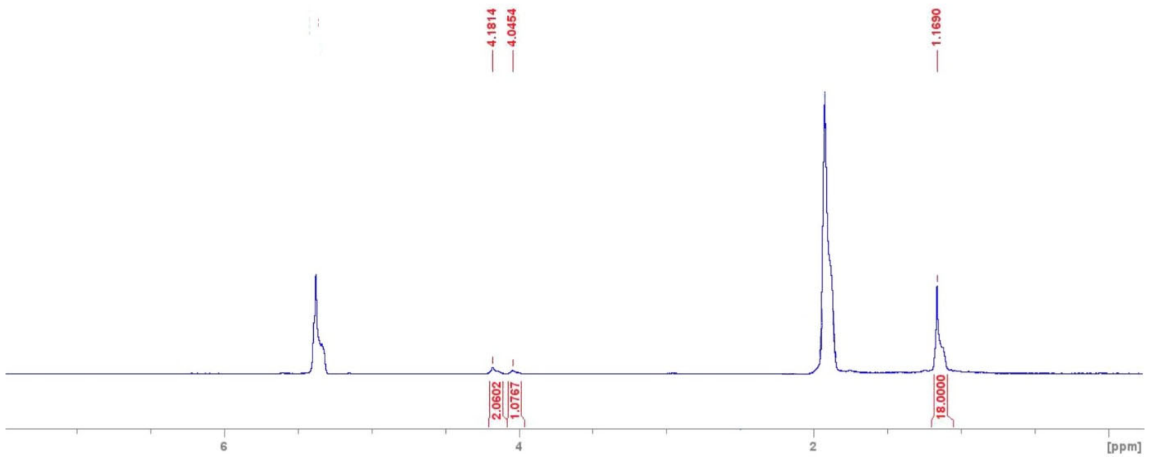

Figure S7 ^{1}H NMR (400.3 MHz, CD_2Cl_2/CD_3CN) spectrum of polymer **5**.

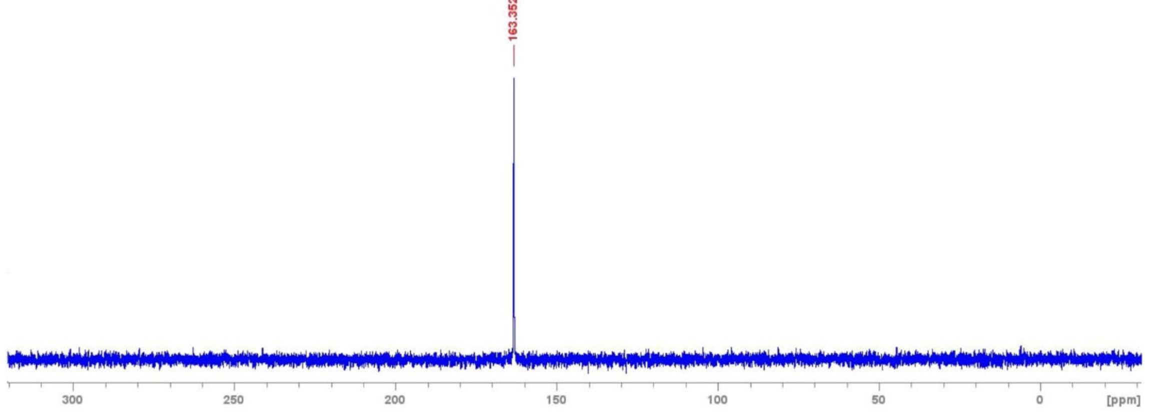

Figure S8 $^{31}P\{^{1}H\}$ NMR (162.0 MHz, CD_2Cl_2/CD_3CN) spectrum of polymer **5**.

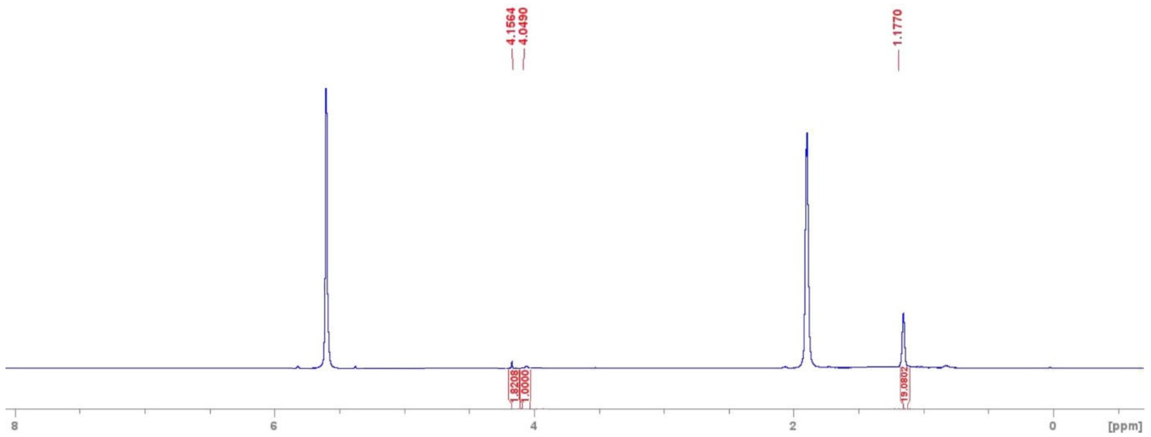

Figure S9 ^{1}H NMR (400.3 MHz, CD_2Cl_2/CD_3CN) spectrum of polymer **6**.

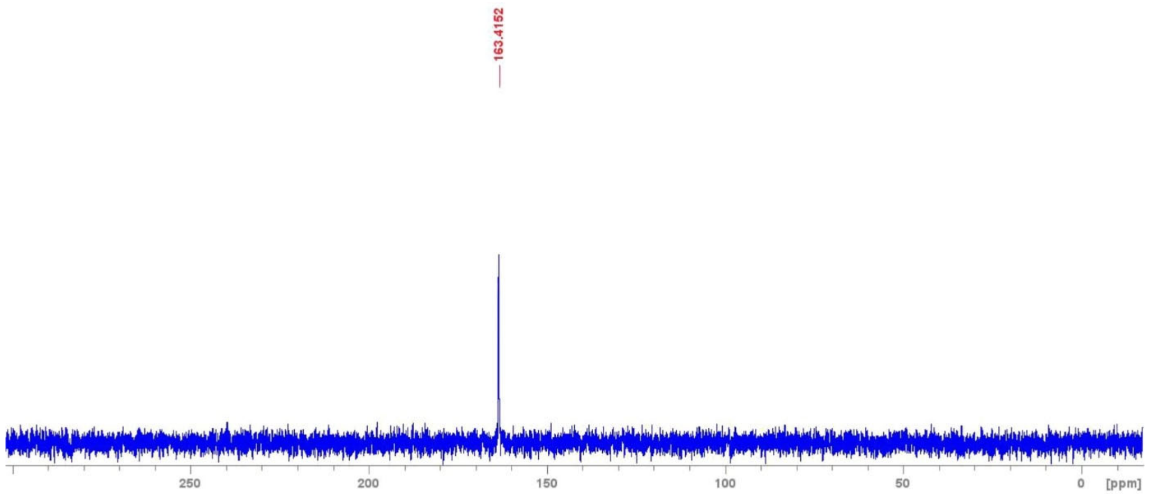

Figure S10 $^{31}P\{^{1}H\}$ NMR (162.0 MHz, CD_2Cl_2/CD_3CN) spectrum of polymer **6**.

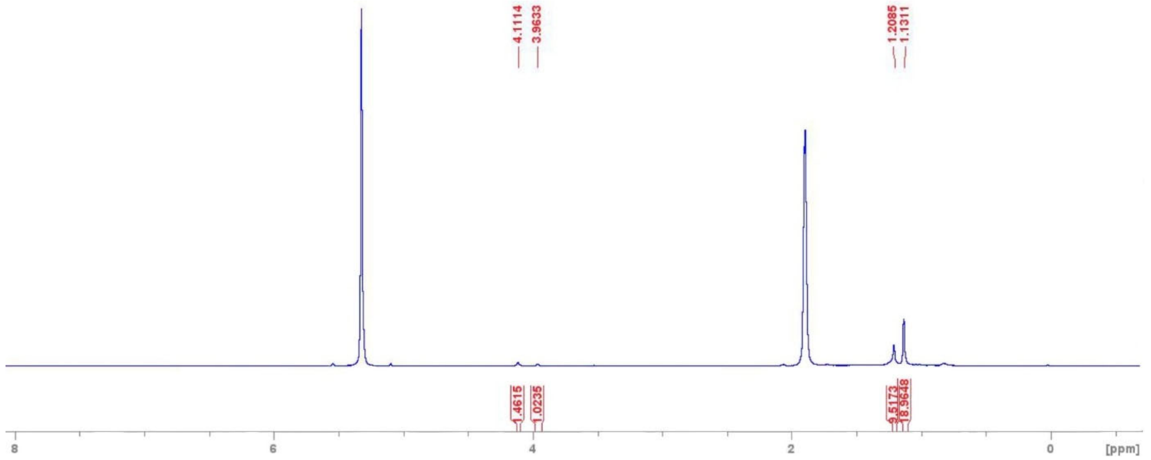

Figure S11 ^{1}H NMR (400.3 MHz, CD_2Cl_2/CD_3CN) spectrum of polymer **7**.

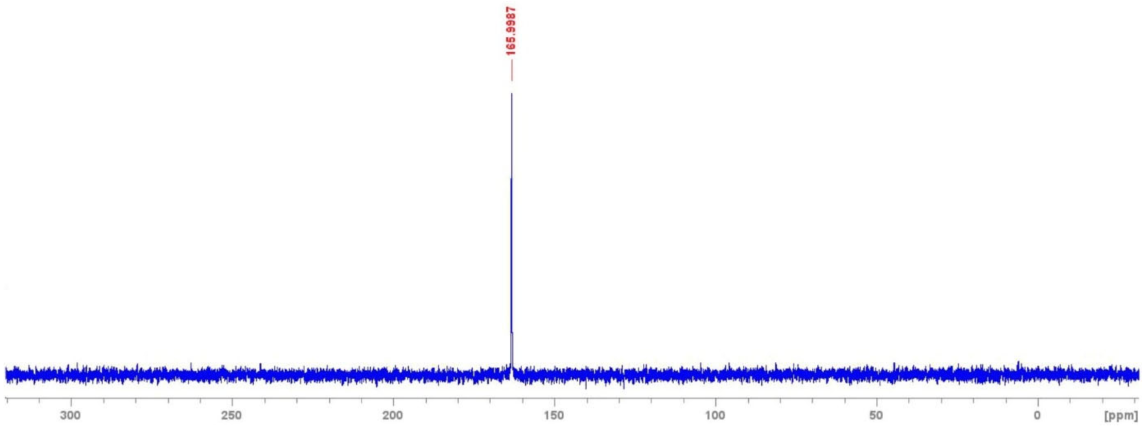

Figure S12 $^{31}P\{^{1}H\}$ NMR (162.0 MHz, CD_2Cl_2/CD_3CN) spectrum of polymer **7**.

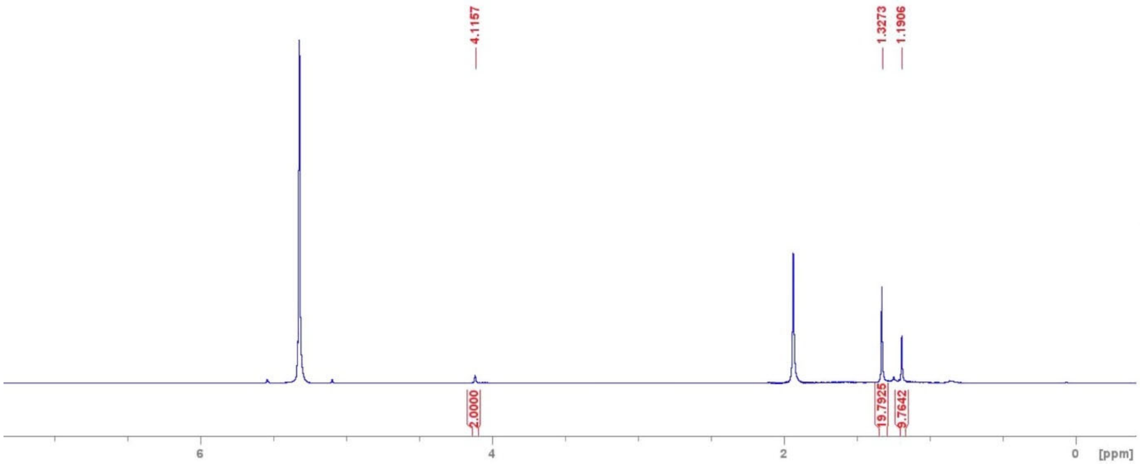

Figure S13 ^{1}H NMR (400.3 MHz, CD_2Cl_2/CD_3CN) spectrum of polymer **8**.

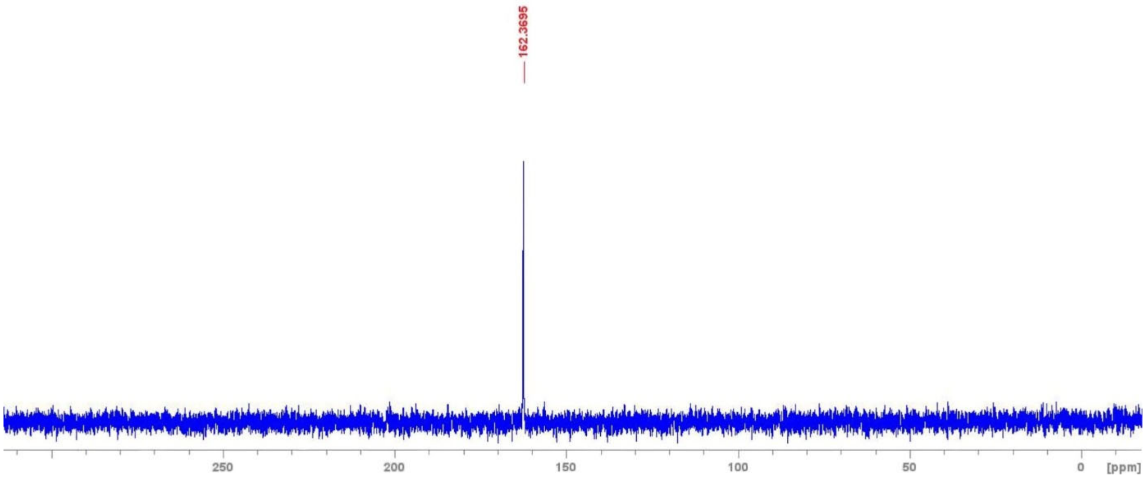

Figure S14 $^{31}P\{^{1}H\}$ NMR (162.0 MHz, CD_2Cl_2/CD_3CN) spectrum of polymer **8**.

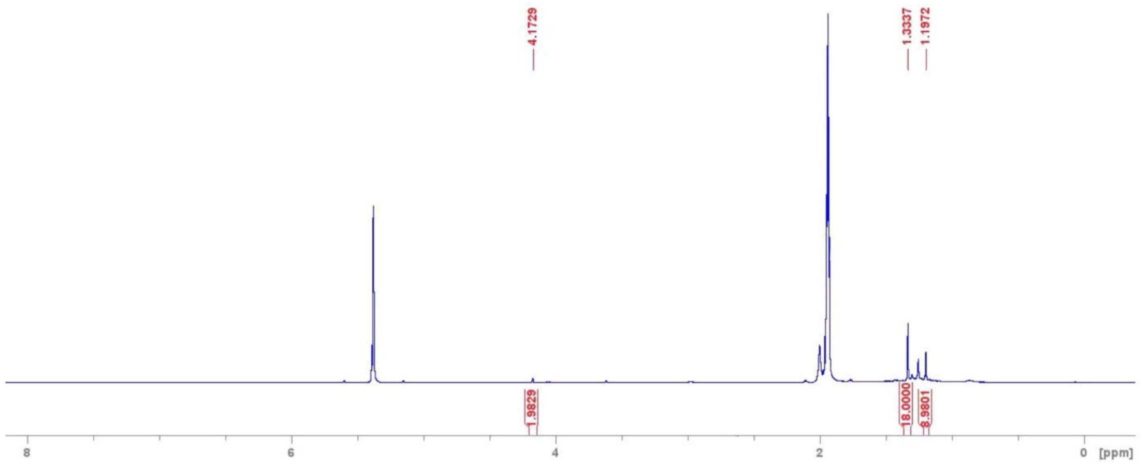

Figure S15 ^{1}H NMR (400.3 MHz, CD_2Cl_2/CD_3CN) spectrum of polymer **9**.

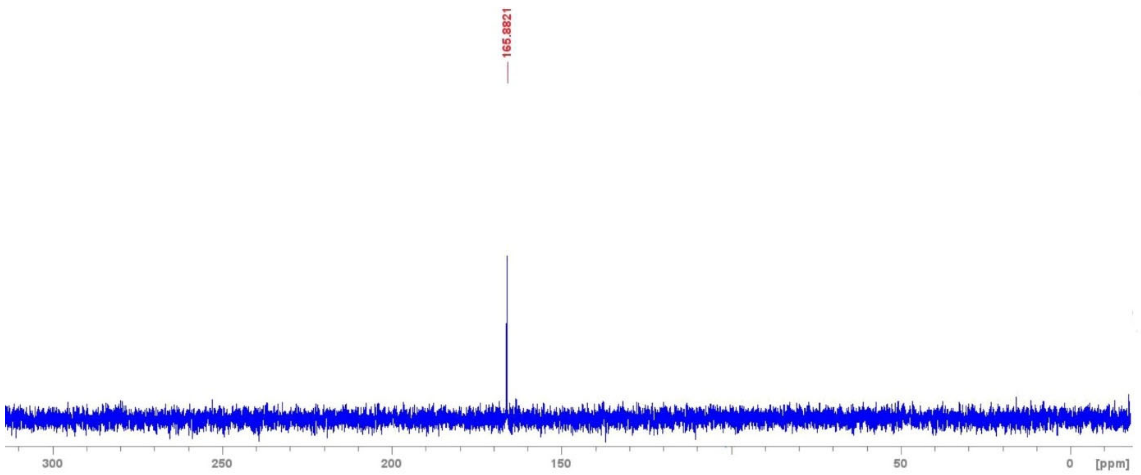

Figure S16 $^{31}P\{^{1}H\}$ NMR (162.0 MHz, CD_2Cl_2/CD_3CN) spectrum of polymer **9**.

3. Mass Spectra:

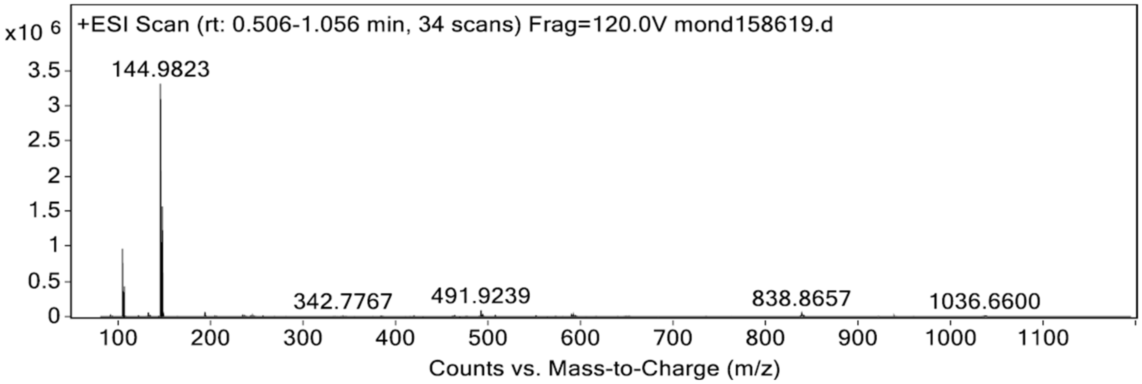

Figure S17 ESI-MS spectrum of polymer **2**.

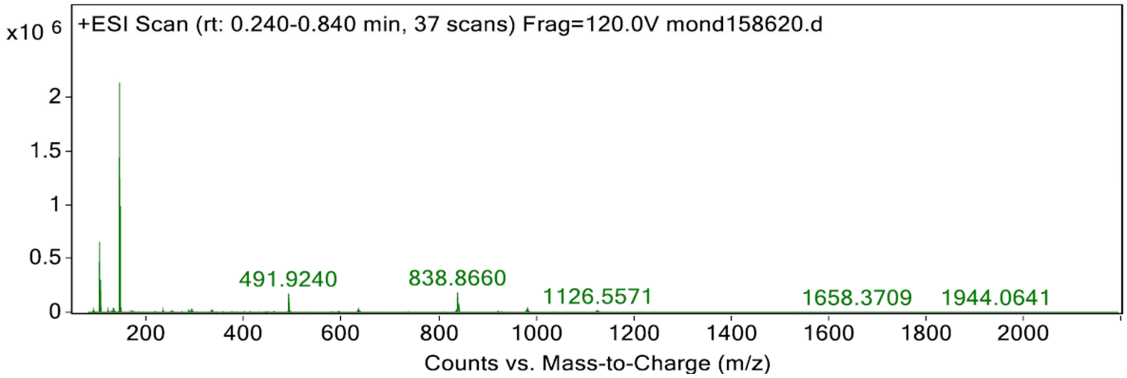

Figure S18 ESI-MS spectrum of polymer **3**.

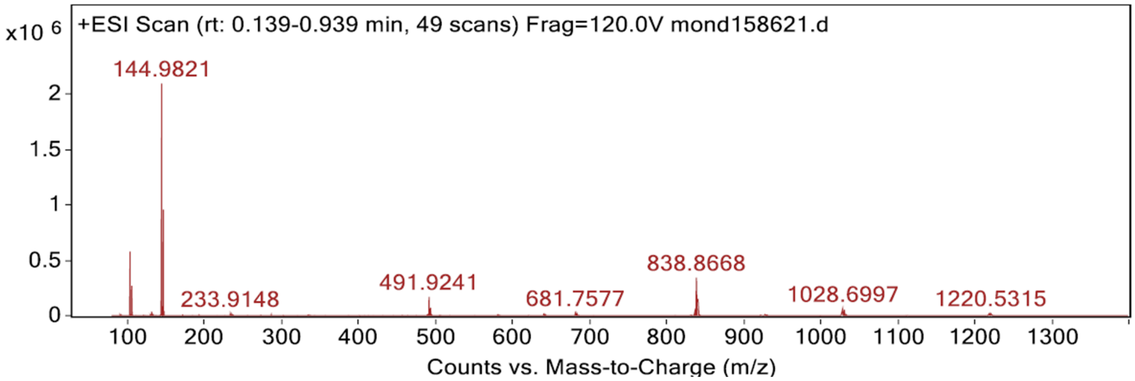

Figure S19 ESI-MS spectrum of polymer **4**.

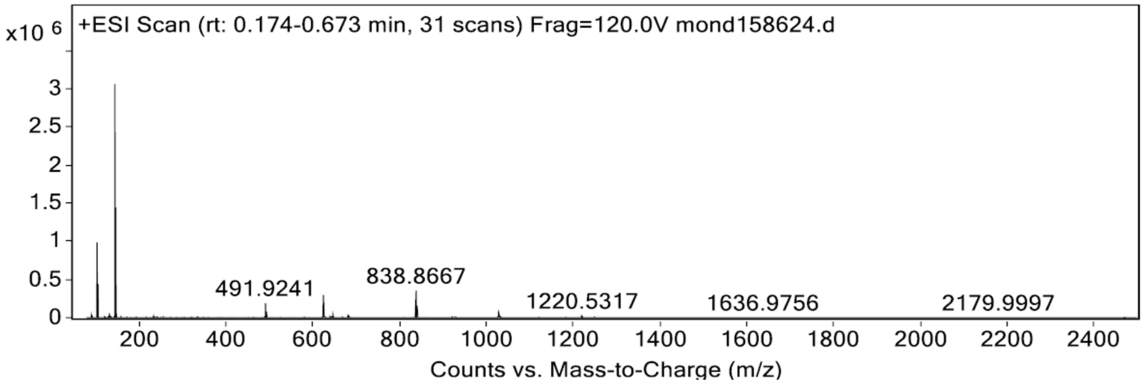

Figure S20 ESI-MS spectrum of polymer **5**.

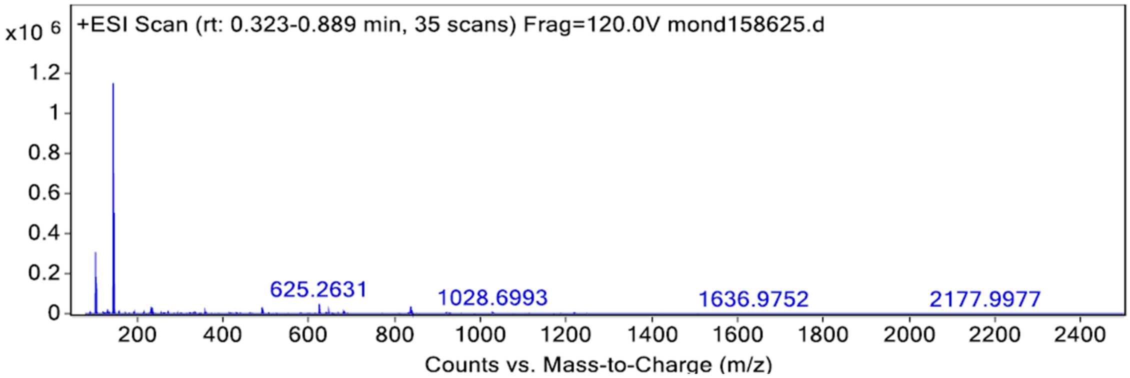

Figure S21 ESI-MS spectrum of polymer **6**.

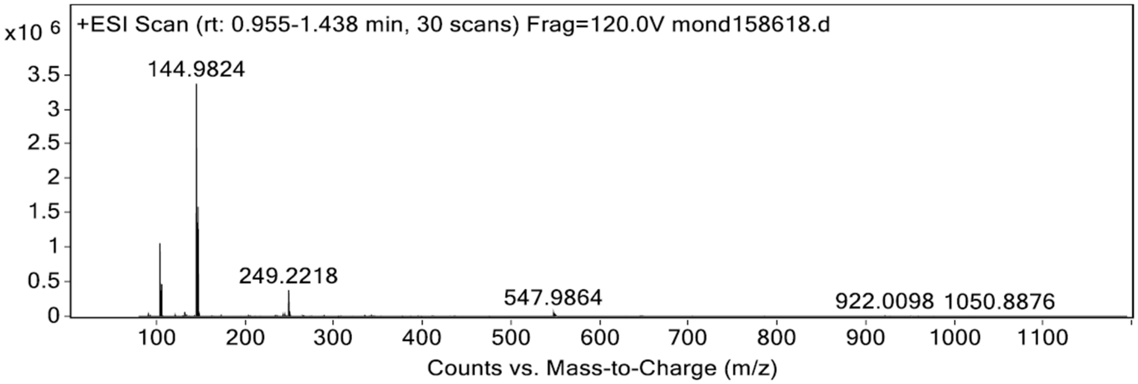

Figure S22 ESI-MS spectrum of polymer **7**.

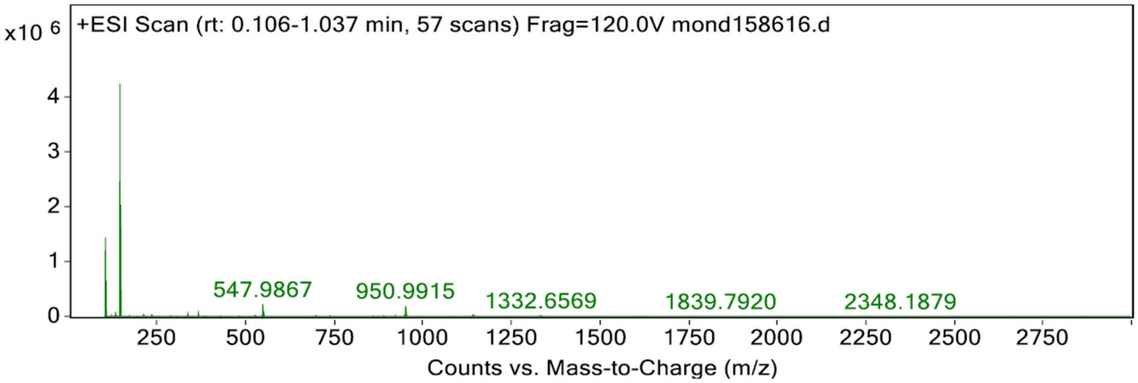

Figure S23 ESI-MS spectrum of polymer **8**.

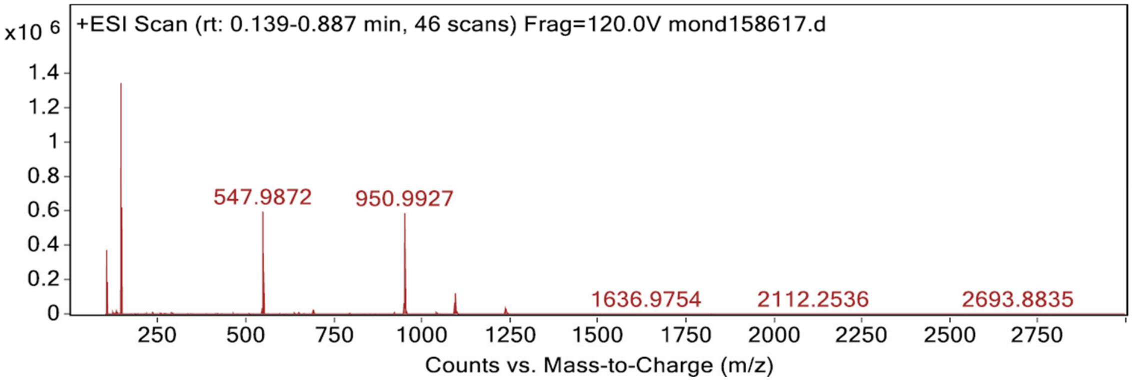

Figure S24 ESI-MS spectrum of polymer **9**.

4. Computational Details:

Gas phase geometry optimizations of the cationic fragments (the calculations did not account for the anionic part) were carried out using Gaussian 09 (rev. E.01) series of program[5] with the B3LYP functional[6] in conjunction with def2-TZVP[7] basis set. Note that all geometry optimizations were performed at the B3LYP/TZVP level of theory without inclusion of an empirical dispersion correction. This level of theory was adopted consistently throughout the study to understand the qualitative comparison of the electronic structure. The optimized geometries were characterized as true minima via analytical frequency calculations (zero negative eigenvalues of the Hessian).

The molecular orbital calculations (B3LYP/Def2-TZVP) conducted on [Cp^RFeP_5] systems (where Cp^R = Cp*, Cp'', and Cp''') reveal how substitution influences the electronic structure. For [Cp^*FeP_5], both the HOMO and LUMO are characterized by double degeneracy. In contrast, introduction of the *ᵗBu* group in [$Cp''FeP_5$] and [$Cp'''FeP_5$] breaks this symmetry, which is evident from alterations in their respective frontier molecular orbitals. Furthermore, the orbital energies for these *tBu*-substituted species are somewhat higher than those for [Cp^*FeP_5].

Despite these differences, [$Cp''FeP_5$] and [$Cp'''FeP_5$] exhibit electronic properties broadly analogous to [Cp^*FeP_5], suggesting similar electronic effects. However, the increased steric bulk from the *tBu* groups is likely to influence the structural organization of the resulting self-assembled products with CuX (X = Cl, Br, I). Thus, while electronic effects remain comparable, steric factors introduced by *ᵗBu* substitution may have a notable impact on the assembly outcomes.

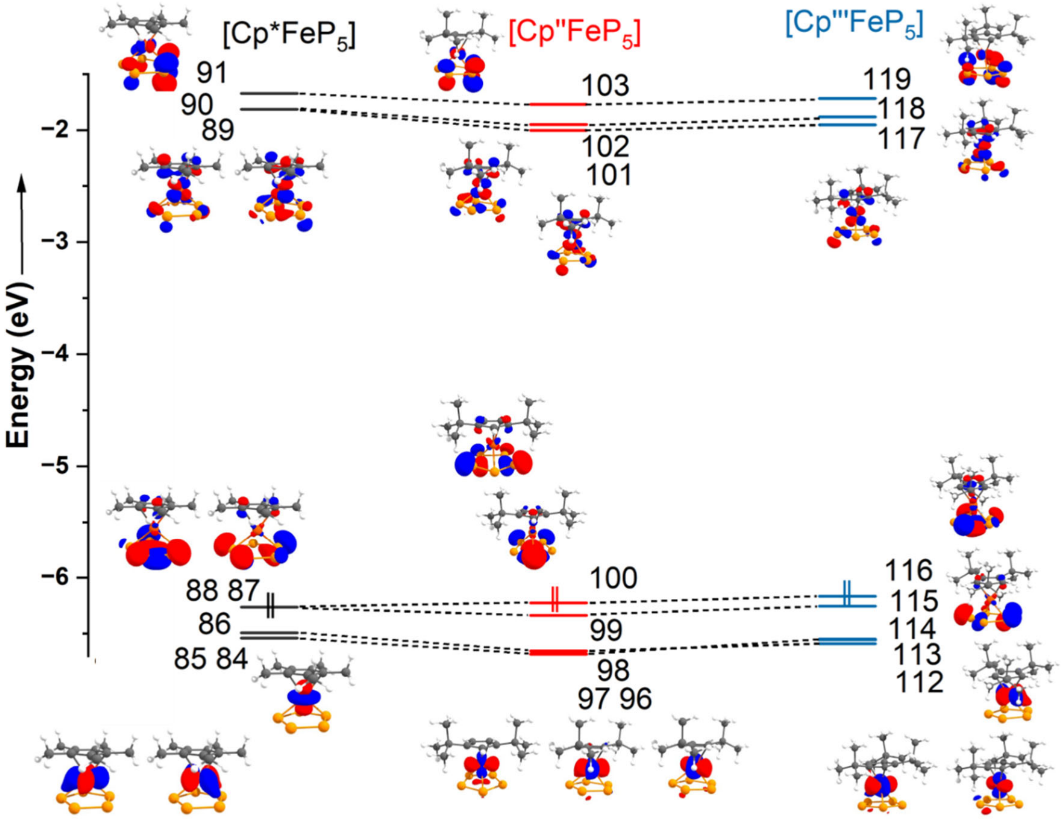

Figure S25 Frontier molecular orbitals of [Cp^*FeP_5], [$Cp''FeP_5$] and [$Cp'''FeP_5$].

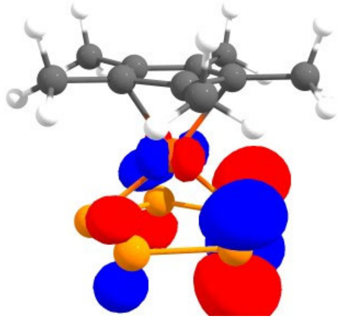

LUMO+2 (91, -1.660 eV)

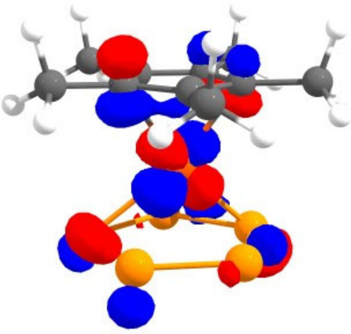

LUMO+1 (90, -1.799 eV)

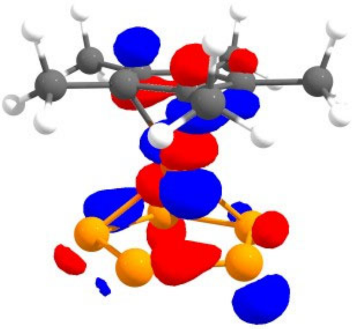

LUMO (89, -1.800 eV)

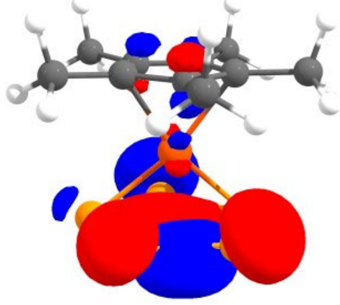

HOMO (88, -6.249 eV)

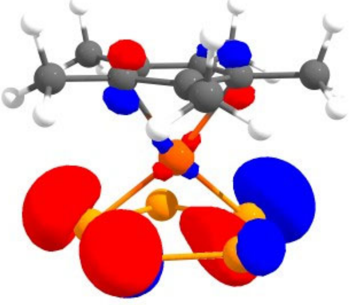

HOMO-1 (87, -6.250 eV)

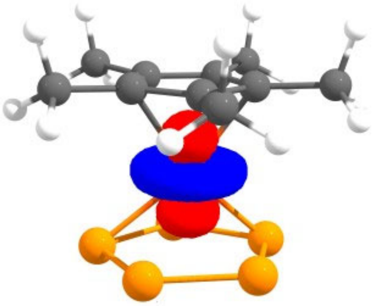

HOMO-2 (86, -6.250 eV)

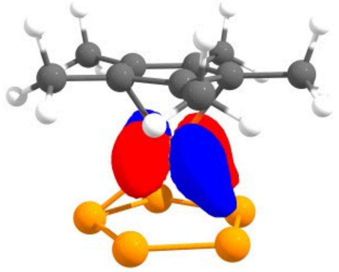

HOMO-3 (85, -6.526 eV)

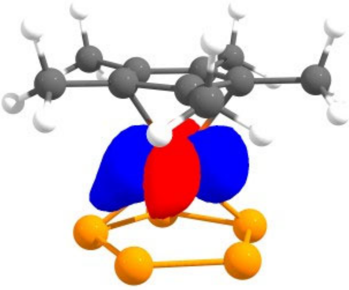

HOMO-4 (84, -6.527 eV)

Figure S26 Frontier molecular orbitals and related energy values of [Cp*FeP$_5$].

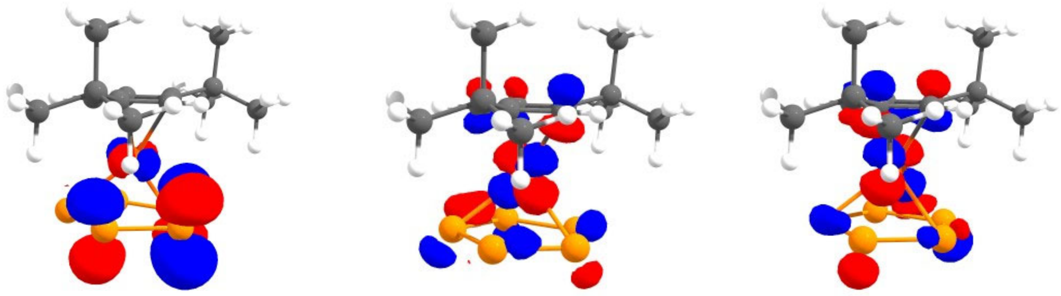

LUMO+2 (103, -1.758 eV) **LUMO+1 (102, -1.937 eV)** **LUMO (101, -1.990 eV)**

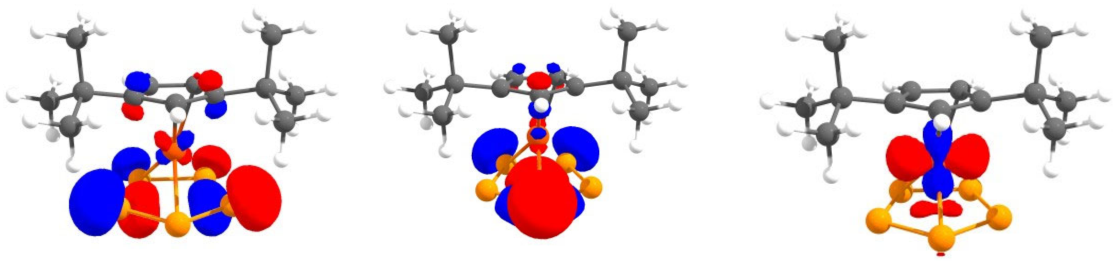

HOMO (100, -6.210 eV) **HOMO-1 (99, -6.323 eV)** **HOMO-2 (98, -6.639 eV)**

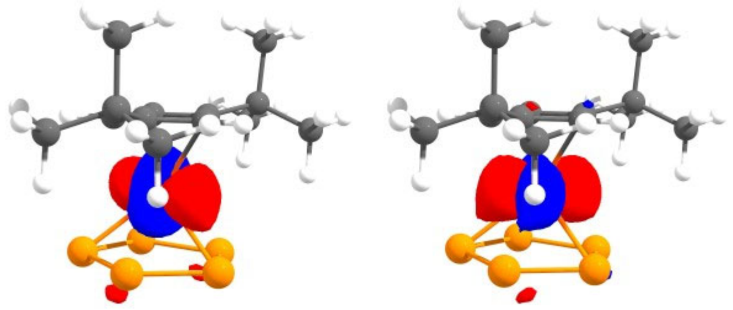

HOMO-3 (97, -6.667 eV) **HOMO-4 (96, -6.669 eV)**

Figure S27 Frontier molecular orbitals and related energy values of [Cp''FeP_5].

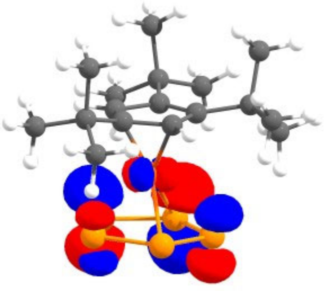

LUMO+2 (119, -1.704 eV)

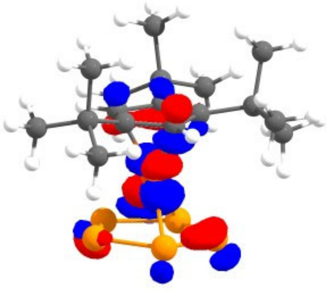

LUMO+1 (118, -1.868 eV)

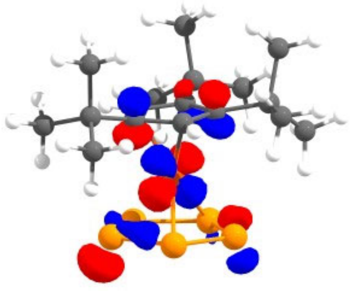

LUMO (117, -1.938 eV)

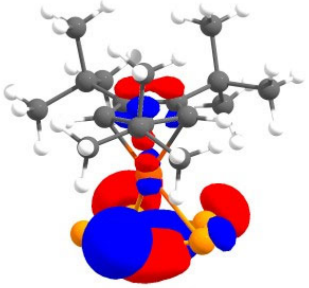

HOMO (116, -6.153 eV)

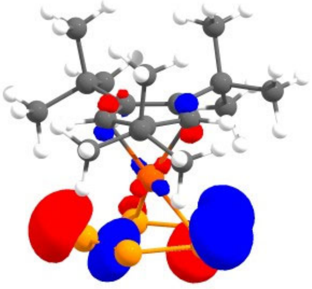

HOMO-1 (115, -6.239 eV)

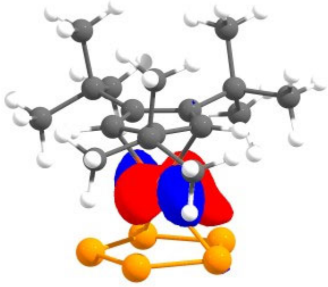

HOMO-2 (114, -6.535 eV)

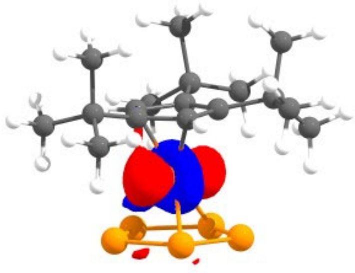

HOMO-3 (113, -6.542 eV)

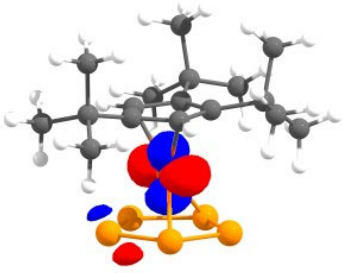

HOMO-4 (112, -6.574 eV)

Figure S28 Frontier molecular orbitals and related energy values of [Cp'''FeP_5].

XYZ Coordinates of the optimized structures

Cp*FeP_5

E(HF) =-3361.0318139

26	-0.086410000	-0.000241000	-0.000539000
15	-1.678654000	1.806295000	0.028966000
15	-1.678998000	0.529541000	1.726573000
15	-1.679027000	-1.479749000	1.036740000
15	-1.678346000	0.586151000	-1.709976000
15	-1.677506000	-1.444778000	-1.087153000
6	1.648766000	-0.402758000	1.147850000
6	1.648982000	0.966459000	0.738966000
6	1.650436000	1.001012000	-0.689467000
6	1.649815000	-1.214344000	-0.028050000
6	1.650685000	-0.346839000	-1.163519000
6	1.733970000	2.152996000	1.645487000
1	1.256820000	3.029794000	1.209273000
1	2.781001000	2.407203000	1.839206000
1	1.257404000	1.961991000	2.606140000
6	1.737233000	2.229143000	-1.537920000
1	1.251577000	2.087678000	-2.502680000
1	2.784406000	2.485773000	-1.727725000
1	1.269028000	3.086147000	-1.055330000
6	1.734534000	-0.898644000	2.555916000
1	1.273231000	-0.203730000	3.256369000
1	2.781413000	-1.022176000	2.851212000
1	1.241542000	-1.862863000	2.674337000
6	1.740700000	-0.774155000	-2.593703000
1	1.263289000	-1.739571000	-2.757151000
1	2.788806000	-0.866910000	-2.896071000
1	1.267329000	-0.054957000	-3.260909000
6	1.739047000	-2.706580000	-0.063902000
1	1.265894000	-3.159940000	0.806316000
1	2.787003000	-3.023114000	-0.074332000
1	1.260895000	-3.118696000	-0.951855000

Cp"FeP_5

E (HF) =-3478.9898798

26	0.000024000	-0.473643000	-0.177755000
15	-1.059943000	-2.381928000	-1.221530000
15	1.711086000	-1.928040000	0.747249000
15	1.061679000	-2.381237000	-1.221193000
15	-1.710362000	-1.929189000	0.746644000
15	0.000093000	-1.606131000	1.958635000
6	-1.169429000	1.329437000	-0.285650000
6	-0.000349000	1.532160000	0.505307000
1	-0.000484000	1.828037000	1.540482000
6	-0.709988000	0.922947000	-1.571494000
1	-1.330113000	0.656968000	-2.411014000
6	0.709561000	0.923209000	-1.571471000
1	1.329691000	0.657417000	-2.411031000
6	-3.643847000	0.978388000	-0.728452000
1	-3.614602000	-0.089959000	-0.517958000
1	-3.512079000	1.120604000	-1.802386000
1	-4.639647000	1.345213000	-0.471444000
6	-2.586911000	1.744124000	0.082022000
6	-2.873914000	1.576483000	1.582390000
1	-3.875504000	1.949002000	1.806499000
1	-2.171554000	2.142766000	2.196391000
1	-2.828688000	0.531345000	1.886311000
6	2.586120000	1.745155000	0.082022000
6	2.873205000	1.577580000	1.582391000
1	2.828650000	0.532379000	1.886180000
1	2.170506000	2.143340000	2.196432000
1	3.874570000	1.950728000	1.806461000
6	3.643368000	0.979880000	-0.728422000
1	4.638989000	1.347543000	-0.471885000
1	3.511229000	1.121455000	-1.802393000
1	3.614977000	-0.088420000	-0.517477000
6	-2.695487000	3.247776000	-0.271926000
1	-2.512224000	3.417565000	-1.334451000
1	-1.975495000	3.841226000	0.294547000
1	-3.697286000	3.614049000	-0.036716000
6	2.693936000	3.248860000	-0.271964000
1	1.973451000	3.841889000	0.294313000
1	2.510828000	3.418487000	-1.334551000
1	3.695464000	3.615711000	-0.036556000
6	1.168806000	1.329876000	-0.285620000

Cp'''FeP_5

E (HF) =-3636.275835

26	-0.479276000	-0.491291000	-0.011045000
15	-1.294335000	-1.844212000	-1.857670000
15	-2.774206000	-1.101371000	-0.541774000
15	-2.147813000	-1.481746000	1.450504000
15	0.251609000	-2.699876000	-0.680676000
15	-0.300637000	-2.504180000	1.358942000
6	-0.046400000	1.192565000	1.162500000
1	-0.366673000	1.294513000	2.183000000
6	-0.030305000	1.257665000	-1.086028000
1	-0.332900000	1.422225000	-2.104220000
6	1.215950000	0.669535000	-0.697491000
6	1.206758000	0.628774000	0.758269000
6	-0.794180000	1.633705000	0.043080000
6	3.431100000	-0.497910000	-1.490061000
1	4.051298000	-0.206758000	-0.646741000
1	4.085475000	-0.550214000	-2.362454000
1	3.040795000	-1.499550000	-1.311612000
6	2.301961000	0.347810000	1.813774000
6	-1.988012000	2.579302000	0.065418000
6	2.304771000	0.503172000	-1.782492000
6	-2.749715000	2.586305000	-1.268889000
1	-2.107104000	2.883365000	-2.099674000
1	-3.568528000	3.306747000	-1.218402000
1	-3.175647000	1.609218000	-1.495077000
6	3.405889000	1.417463000	1.648801000
1	3.969278000	1.293752000	0.727138000
1	4.112073000	1.345559000	2.478887000
1	2.981658000	2.423650000	1.654607000
6	-2.967391000	2.274163000	1.209132000
1	-3.465417000	1.316461000	1.067017000
1	-3.735514000	3.049377000	1.250058000
1	-2.468977000	2.255709000	2.179556000
6	2.931727000	1.905308000	-1.989592000
1	2.178167000	2.630088000	-2.301981000
1	3.695110000	1.856120000	-2.769489000
1	3.401958000	2.282236000	-1.082282000
6	1.734951000	0.521078000	3.239222000
1	1.412770000	1.544761000	3.435748000
1	2.518360000	0.282365000	3.960249000
1	0.898835000	-0.153149000	3.428134000
6	2.931374000	-1.057640000	1.781160000
1	2.225068000	-1.803612000	2.139822000
1	3.797827000	-1.074682000	2.446922000
1	3.262714000	-1.363928000	0.797300000
6	-1.397025000	3.992634000	0.294481000
1	-0.878302000	4.054513000	1.252783000
1	-2.197243000	4.736023000	0.293053000
1	-0.687244000	4.256733000	-0.491702000
6	1.694833000	0.073348000	-3.134426000
1	1.229322000	-0.908814000	-3.074172000
1	2.492161000	0.020956000	-3.877898000
1	0.954861000	0.777003000	-3.512458000

References

1. CrysAlisPro Software System, Rigaku Oxford Diffraction/Rigaku Corporation, Oxford, UK.
2. O. V. Dolomanov, L. J. Bourhis, R. J. Gildea, J. A. K. Howard and H. Puschmann, *Olex2: a complete structure solution, refinement and analysis program*, *J. Appl. Crystallogr.*, **2009**, *42*, 339–341.
3. G. M. Sheldrick, *SHELXT – Integrated space-group and crystal-structure determination*, *Acta Crystallogr., Sect. A*, **2015**, *71*, 3–8.
4. G. M. Sheldrick, *Crystal structure refinement with SHELXL*, *Acta Crystallogr., Sect. C*, **2015**, *71*, 3–8.
5. M. J. Frisch, G. W. Trucks, H. B. Schlegel, G. E. Scuseria, M. A. Robb, J. R. Cheeseman, G. Scalmani, V. Barone, B. Mennucci, G. A. Petersson, H. Nakatsuji, M. Caricato, X. Li, H. P. Hratchian, A. F. Izmaylov, J. Bloino, G. Zheng, J. L. Sonnenberg, M. Hada, M. Ehara, K. Toyota, R. Fukuda, J. Hasegawa, M. Ishida, T. Nakajima, Y. Honda, O. Kitao, H. Nakai, T. Vreven, J. A. Montgomery, Jr., J. E. Peralta, F. Ogliaro, M. Bearpark, J. J. Heyd, E. Brothers, K. N. Kudin, V. N. Staroverov, T. Keith, R. Kobayashi, J. Normand, K. Raghavachari, A. Rendell, J. C. Burant, S. S. Iyengar, J. Tomasi, M. Cossi, N. Rega, J. M. Millam, M. Klene, J. E. Knox, J. B. Cross, V.

Bakken, C. Adamo, J. Jaramillo, R. Gomperts, R. E. Stratmann, O. Yazyev, A. J. Austin, R. Cammi, C. Pomelli, J. W. Ochterski, R. L. Martin, K. Morokuma, V. G. Zakrzewski, G. A. Voth, P. Salvador, J. J. Dannenberg, S. Dapprich, A. D. Daniels, O. Farkas, J. B. Foresman, J. V. Ortiz, J. Cioslowski, and D. J. Fox, Gaussian, Inc., Wallingford CT, **2013**.

6. a) A. D. Becke, *J. Chem. Phys.* 1993, **98**, 5648; b) C. Lee, W. Yang and R. G. Parr*, Phys. Rev. B* 1988, **37**, 785-789, c) S. H. Vosko, L. Wilk and M. Nusair, *Can. J. Phys.* 1980, **58,** 1200; d) P. J. Stephens, F. J. Devlin, C. F. Chabalowski and M. J. Frisch, *J. Phys. Chem*. 1994, **98**, 11623.
7. Weigend F.; Ahlrichs, R. Balanced basis sets of split valence, triple zeta valence and quadruple zeta valence quality for H to Rn: Design and assessment of accuracy. *Phys. Chem. Chem. Phys.* **2005**, *7*, 3297-3305.
